# Supplementary material for: Feasibility, usability and acceptability of paediatric lung ultrasound among healthcare providers and caregivers for the diagnosis of childhood pneumonia in resource-constrained settings: a qualitative study
Source: BMJ Open. 2021 Mar 11;11(3):e042547. doi: 10.1136/bmjopen-2020-042547 (PMC7957133; doi:10.1136/bmjopen-2020-042547)
Supplement: Supplementary data [file bmjopen-2020-042547supp001.pdf]

PLUS Healthcare Provider/Administrator Interview Guide

**Administrative Information**	
**Participant ID number: PLUS-5 __ __ __**	
**Does the interviewee agree to be audio recorded during the interview?**	☐ Yes ☐ No
**Date written informed consent (IC) signed:** |D|D| - |M|M|M| - |Y|Y|Y|Y|	
**Consent form signed prior to any study questions?**	☐ Yes ☐ No
**Name of the person who explained the IC:**	
**In which language was the IC explained?**	☐ Portuguese ☐ Changana ☐ English
**Date of interview:** |D|D| - |M|M|M| - |Y|Y|Y|Y|	
**Location of interview:**	
**Was the interview audio-recorded?**	☐ Yes ☐ No
**Start time:** |H|H| : |M|M|	**End time:** |H|H| : |M|M|
**Name of interviewer:**	

Instruction for research team members

- Use this document as a guide to the interview with a healthcare provider/administrator.
- Conduct the interview in the language with which the interviewee feels most comfortable.
- Prior to beginning the interview, show the healthcare provider/administrator the lung ultrasound probe and tablet. Let the provider/administrator hold and explore the tablet, application and probe.
- Please introduce each question separately. The interview must flow as a conversation. If you notice that the interviewee is hesitant in answering, does not give an in-depth response, or the response is not satisfactory, please probe or ask follow-up questions, but do NOT prompt any specific answer. Several probes are suggested, but you may also ask follow-up questions that are not listed in this guide but are necessary for the complete expression of the interviewee's views.
- Please record the proceedings using the tape recorder/digital recorder (if consent is provided) and state the Participant ID number. Please also record the verbatim responses in appropriate boxes.
- All responses must be treated with confidentiality. Do not discuss or share the responses with anyone outside of the PLUS study team.

A. Demographic information & background

Age:

Gender:

Highest level of education completed:

Years of medical education/training:

Type of medical education/training received (e.g., medical doctor, technician, nurse):

Riaz A, *et al. BMJ Open* 2021; 11:e042547. doi: 10.1136/bmjopen-2020-042547

Duration of employment at [name of facility]:

Duration of employment in current role:

Years of medical experience after training completed:

B. Role of healthcare provider / administrator

How would you describe your role here at [name of facility]?

What are your responsibilities?

Did you work in the medical field prior to working here? If yes, please describe.
Probes: length of time worked, responsibilities

Are you involved in patient care? Please describe your roles and responsibilities.

Are you involved in clinical research? Please describe your roles and responsibilities.

Riaz A, *et al. BMJ Open* 2021; 11:e042547. doi: 10.1136/bmjopen-2020-042547

Do you ever contribute to the policy development for the care and treatment of children at this facility? Please describe.
**C.** Environment
What are the most common reasons that caregivers bring their children to [facility name]?<br><br>If a caregiver does not bring his/her child here, what are the other options for seeking care?<br>• Probe for other facilities and other types of healthcare providers such as traditional healers, faith-based healers, drug shops, etc.<br>• Why might they seek care at another facility or with another type of healthcare provider?
What are the current constraints to providing care to children at [facility name]? Please explain.<br>• What makes providing care more difficult?<br>• What makes it easier?

D. Pneumonia diagnosis

The questions in Part D may not be appropriate for all interviewees. They may be skipped at the discretion of the local principal investigator or designee.

Tell me about the use of the Integrated Management of Childhood Illnesses (IMCI) guidelines at this facility. Do healthcare providers use IMCI? If yes, how do they use them?

If they don't use IMCI, why not? What do they use to guide diagnosis and treatment?

For pneumonia specifically, tell me about the role of IMCI guidelines in diagnosis. If IMCI guidelines are used, please describe. If IMCI guidelines are not used, how is pneumonia diagnosed?

Tell me about the use of chest x-ray at this facility.

Tell me about its role in diagnosing pneumonia.

When are chest x-rays used as a diagnostic tool for children? How is the decision made to order and use a chest x-ray in the care of a child?

What constraints have providers at [name of facility] faced in using x-ray in caring for children?
**E.** Use of technology
What technologies are used for patient care at [name of facility]? Please describe the technologies and their use.
Are any handheld or portable devices used for patient care at [name of facility]? If so, please describe.<br>• Smartphones?<br>• Tablets?<br>• Other mHealth applications?<br><br>How are these devices stored and maintained?<br>• Are they shared between providers?<br>• How are they kept secure?<br>• How are they cleaned?
Probe on how involved or familiar the respondent is with the PLUS study and its procedures/purpose. Aside from the PLUS study, is ultrasound used in any capacity at [name of facility]?

IF YES, for what? How frequently?
**F.** Experience with ultrasound<br>*This section is for clinical care providers only. If the interviewee is not a clinical care provider, please skip to Part G.*
Tell me about your experience with ultrasound? Have you performed it? Seen it performed?<br><br>If you have performed ultrasound, for what condition/reason? How long have you been performing ultrasound? How frequently do you do ultrasound examinations?<br><br>Now, tell me about your experience with **lung** ultrasound. Have you performed it? Seen it performed? Were you familiar with lung ultrasound prior to this study?
If you have performed lung ultrasound, describe your experience doing so.<br>• How long have you been performing lung ultrasound?<br>• How frequently do you do lung ultrasound examinations?

- What has gone well while in performing ultrasound examinations? What has gone poorly? Have any problems occurred during an ultrasound exam?
- What aspects of lung ultrasound are easy to use? What aspects are difficult to use?

If you have performed lung ultrasound, how much time (in minutes) did a typical lung ultrasound examination take? What factors made exams go more quickly? More slowly?

- How much time was spent describing the exam to the caregiver?
- How much time was spent setting up the exam on the tablet?

If you have performed lung ultrasound, did you interpret the lung ultrasound exams? If so, tell me about your experience.

- What was difficult about it?
- What was easy about it? What could have made it easier?

How much time (in minutes) did it take to interpret a typical lung ultrasound exam?

- What factors made this process go more quickly? More slowly?

If you did not interpret the lung ultrasound exam, why not? Who did the interpretation?

G. Lung ultrasound training

If you have performed lung ultrasound, how much training (hours/days) did you receive? Please describe this training.

- Who provided this training?
- What aspects of lung ultrasound were easy to learn? What aspects were difficult?

If you have not used lung ultrasound previously, do you think that you could learn how to use the device and perform lung ultrasound exams? Please explain.

How much training (hours and/or days) do you think that this would take?

What barriers would there be to learning how to use the ultrasound device?

Riaz A, *et al. BMJ Open* 2021; 11:e042547. doi: 10.1136/bmjopen-2020-042547

H. Overall feasibility, usability and acceptability of lung ultrasound

What do you like about the lung ultrasound system as a whole? What do you dislike?

What do you like about the tablet? What do you dislike?

- Screen/display

What do you like about the application? What do you dislike?

- Usability/navigation/interface
- Screen/display

What do you like about the probe? What do you dislike?

What would you change to improve this ultrasound system?

Do you think that lung ultrasound could be integrated into this facility? What would this look like?

What would be some of the facilitators of integrating lung ultrasound?

What would be some of the challenges/barriers?

What needs to happen order to introduce lung ultrasound successfully?

- Probes: Ease of use during a patient visit, integration into the current hospital flow operationally

How do you think that the lung ultrasound would impact your ability to care for children?

- What would be improved by it?
- What would be difficult about it?

How do you think that caregivers would respond to the incorporation of lung ultrasound into care at [name of facility]? Please explain.

How do you think decision-makers at local, district and national levels would react to a recommendation involving the use of lung ultrasound for pneumonia diagnosis?

Are there scenarios where you think lung ultrasound should not be used? Please explain.

Do you foresee any problems with keeping the ultrasound or tablet in the hospital when not in use?

- Probes: Losing ultrasound probe or tablet, theft

What level of healthcare provider do you think should perform lung ultrasound exams? Please explain.

What level of healthcare provider do you think should interpret lung ultrasound exams? Please explain.

Riaz A, *et al. BMJ Open* 2021; 11:e042547. doi: 10.1136/bmjopen-2020-042547

Do you think that the same person should perform and interpret the lung ultrasound exams? Please explain.
What do you think would be an appropriate price for a lung ultrasound device? Please explain.
**I.** Closing
Do you have any final comments about the ultrasound device that we did not talk about?<br><br>Do you have any comments on the overall PLUS study that we did not talk about?
Do you have any suggestions for other people we should speak to at [name of facility]?
**"Thank you very much for your time and for all of the helpful information you have provided"**

PLUS Caregiver Qualitative Sub-Study

**Administrative information**
**Participant ID number: PLUS-1 __ __ __** *(Use child's study ID from the main PLUS study)*
**Date written informed consent (IC) signed:** |D|D| - |M|M|M| - |Y|Y|Y|Y|
**Caregiver consent form signed prior to any study questions?** ☐ Yes ☐ No
**Name of the person who explained the IC:**
**In which language was the IC explained?** ☐ Portuguese ☐ Changana ☐ English
**Relationship to child of the person signing IC:** ☐ Mother ☐ Father ☐ Other, specify:
**Was an impartial witness necessary?** ☐ Yes ☐ No<br>**If YES, did the impartial witness sign the consent form?** ☐ Yes ☐ No
**Does the caregiver agree to be audio recorded** ☐ Yes ☐ No

PLUS Caregiver Direct Observation Guide

**Name of observer:**
**Ultrasound exam start time:** |H|H| : |M|M| **Ultrasound exam end time:** |H|H| : |M|M|
**<u>Instructions for research team members:</u>**<br>• Observe interactions as the study team member explains the ultrasound examination to the caregiver, and during the ultrasound exam itself.<br>• Record observations in the fields below. Any questions and/or comments from the caregiver should be recorded verbatim.<br>• Your role is that of an observer. Avoid interaction with the ultrasound technician or caregiver during the ultrasound exam.<br>• All responses must be treated with confidentiality. Do not discuss or share the responses with anyone outside of the PLUS study team.<br>• Please cross-check the narratives written with the recorded version as a reference, and correct as necessary.
**Approximately how much time (minutes) did research team staff spend explaining the ultrasound exam to the caregiver?**
**Record verbatim any questions/comments by the caregiver during the explanation of the ultrasound exam, as well as the research staff's responses. Provide context as necessary.**

**How did the child react to the ultrasound exam?** Examples: crying, calm
**Did you observe anything that stood out to you?**

PLUS Caregiver In-Depth Interview Guide

**Date of Interview:** |D|D| - |M|M|M| - |Y|Y|Y|Y|
**Start time:** |H|H| : |M|M| **End time:** |H|H| : |M|M|
**Name of interviewer:**

Instructions for research team members:

- Use this document as a guide to the interview with the child's caregiver.
- Conduct the interview in the language with which the caregiver feels most comfortable.
- The interview should take place in a quiet place that allows privacy.
- Please introduce each question separately. The interview must flow as a conversation. If you notice that the caregiver is hesitant in answering, does not give an in-depth response, or the response is not satisfactory, please probe or ask follow-up questions, but do NOT prompt any specific answer. Several probes are suggested, but you may also ask follow-up questions that are not listed in this guide but are necessary for the complete expression of the caregiver's views.
- Please record the proceedings using the tape recorder/digital recorder (if caregiver consent is provided) and state the PLUS Participant ID number. Please also record the verbatim responses in the appropriate boxes.
- Please cross-check the narratives written with the recorded version as a reference, and correct as necessary.
- All responses must be treated with confidentiality. Do not discuss or share the responses with anyone outside of the PLUS study team.

A. Background

Caregiver's age:

Caregiver's gender:

Caregiver's highest level of education completed:

Tell me about (name of the index child), who s/he lives with and who is responsible for caring for him/her.

- Household characteristics (physical, family size, main sources of income)
- Parents, siblings characteristics
- Role of respondent in caretaking (if not the father/mother, why)

B. Visit

Looking at the past 3 months, what was your healthcare use in regard to child health care?

- Probe to help participant estimate his/her use for routine visits, illness episodes, chronic illness follow up, clinical study visits
- Do you consider these (refer to the past three months) as typical for you? (If not, what made this period different from what is usual?)

What makes you decide to bring your child into a health facility?

Do you ever go to another health facility if your child/children get sick? Where is that located?

- Which health facilities did you mostly use?
- Why might you go to another health facility instead of this one?

How long does it take you to get to this health facility? Is there a closer health facility to your home? If so, how long does it take you to get there? Describe the care provided by the facility closest to your home.

C. Acceptability of the tablet

Have you ever seen a healthcare provider use a tablet or smart phone application while caring for your child?

IF YES: Where? For what condition/reason?
What do you think about the use of tablets as tools to help healthcare providers provide care for children? Please explain.<br><br>Do you think that tablets can be helpful in medical care? Please explain.<br><br>Do you trust tablets as tools to support medical care? Please explain.
**D. Acceptability of the ultrasound device**
Before today, had you ever seen a healthcare provider use an ultrasound device? IF YES, where? For what reason?

Riaz A, *et al. BMJ Open* 2021; 11:e042547. doi: 10.1136/bmjopen-2020-042547

Before today, had a healthcare provider ever used an ultrasound device on your child? IF YES, where? For what reason?
What did you think about the ultrasound device as it was being used on your child?<br>Probes: Ultrasound gel, ultrasound probe, tablet, undressing the child, child cries<br><br>What did you like about it/its use?<br><br>What didn't you like about it/its use?
Did the healthcare provider explain what she or he was doing as s/he was using the ultrasound device? If yes, was this helpful?

If no, what did you expect the healthcare provider to tell you when s/he was using the ultrasound device?<br><br>What do you think is the purpose of the ultrasound device?<br>• If illnesses are mentioned, probe for the exact name of the illness being targeted?
Did you have any concerns about the ultrasound device being used during your child's visit?
Did you look at the screen while the ultrasound device was being used on your child?<br><br>Why or why not?<br><br>If yes, what did you see? Did you find this helpful?
What did you think about the length of the ultrasound exam?

Do you think that the ultrasound device should be used in this hospital? Please explain.
Do you see any problems with using this ultrasound device on children that come to this hospital? Please explain.
**E. Comparison to chest x-ray**
Before today's visit, had your child ever had a chest x-ray? *(Research staff to show photo of an x-ray machine)*
What did you think about the use of the ultrasound device compared to the chest x-ray?<br><br>What did you like more? Why?<br><br>What did you like less? Why?
**F. Influence of ultrasound device on care seeking and perceived care**

Do you think that healthcare providers at this hospital should continue to use an ultrasound when evaluating and caring for sick children? Please explain.
If you were choosing between taking your child to a health facility that offers ultrasound and a health facility that does not, would the ultrasound factor into your choice? Please explain.
**G. Closing**
Do you have any final comments about the ultrasound device that we did not talk about?<br><br>Do you have any final comments about the research study that we did not talk about?
**"Thank you very much for your time and for all of the helpful information you have provided"**

Riaz A, *et al. BMJ Open* 2021; 11:e042547. doi: 10.1136/bmjopen-2020-042547
