# Supplementary material for: Feasibility, usability and acceptability of paediatric lung ultrasound among healthcare providers and caregivers for the diagnosis of childhood pneumonia in resource-constrained settings: a qualitative study
Source: BMJ Open. 2021 Mar 11;11(3):e042547. doi: 10.1136/bmjopen-2020-042547 (PMC7957133; doi:10.1136/bmjopen-2020-042547)
Supplement: Author's manuscript [file bmjopen-2020-042547.draft_revisions.pdf]

BMJ Open is committed to open peer review. As part of this commitment we make the peer review history of every article we publish publicly available.

When an article is published we post the peer reviewers' comments and the authors' responses online. We also post the versions of the paper that were used during peer review. These are the versions that the peer review comments apply to.

The versions of the paper that follow are the versions that were submitted during the peer review process. They are not the versions of record or the final published versions. They should not be cited or distributed as the published version of this manuscript.

BMJ Open is an open access journal and the full, final, typeset and author-corrected version of record of the manuscript is available on our site with no access controls, subscription charges or pay-per-view fees (http://bmjopen.bmj.com).

If you have any questions on BMJ Open's open peer review process please email info.bmjopen@bmj.com

BMJ Open

Feasibility, usability, and acceptability of pediatric lung ultrasound among healthcare providers and caregivers for the diagnosis of childhood pneumonia in resource-constrained settings: A qualitative study

Journal:	*BMJ Open*
Manuscript ID	bmjopen-2020-042547
Article Type:	Original research
Date Submitted by the Author:	07-Jul-2020
Complete List of Authors:	Riaz, Atif; Aga Khan University, Pediatrics and Child Health<br>Cambaco, Olga; Centro de Investigação em Saúde de Manhiça<br>Ellington, Laura; University of Washington<br>Lenahan, Jennifer; Save the Children Federation Inc<br>Munguambe, Khatia ; Centro de Investigacao em Saude de Manhica<br>Mehmood, Usma; Aga Khan University, Pediatrics and Child Health<br>Lamorte, Alessandro; Umberto Parini Hospital, Emergency Medicine<br>Qaisar, Sana; Aga Khan University, Pediatrics and Child Health<br>Nisar, Muhammad; Aga Khan University, Pediatrics and Child Health<br>Volpicelli, Giovanni; San Luigi Gonzaga University Hospital, Emergency Medicine<br>Bassat, Quique; ISGLOBAL; Universitat de Barcelona<br>Jehan, Fyezah; Aga Khan University, Paediatrics and Child Health<br>ginsburg, amy sarah; University of Washington, Clinical Trial Center
Keywords:	Paediatric thoracic medicine < PAEDIATRICS, PUBLIC HEALTH, QUALITATIVE RESEARCH, ULTRASONOGRAPHY, Respiratory infections < THORACIC MEDICINE, International health services < HEALTH SERVICES ADMINISTRATION & MANAGEMENT

SCHOLARONE™
Manuscripts

I, the Submitting Author has the right to grant and does grant on behalf of all authors of the Work (as defined in the below author licence), an exclusive licence and/or a non-exclusive licence for contributions from authors who are: i) UK Crown employees; ii) where BMJ has agreed a CC-BY licence shall apply, and/or iii) in accordance with the terms applicable for US Federal Government officers or employees acting as part of their official duties; on a worldwide, perpetual, irrevocable, royalty-free basis to BMJ Publishing Group Ltd ("BMJ") its licensees and where the relevant Journal is co-owned by BMJ to the co-owners of the Journal, to publish the Work in this journal and any other BMJ products and to exploit all rights, as set out in our licence.

The Submitting Author accepts and understands that any supply made under these terms is made by BMJ to the Submitting Author unless you are acting as an employee on behalf of your employer or a postgraduate student of an affiliated institution which is paying any applicable article publishing charge ("APC") for Open Access articles. Where the Submitting Author wishes to make the Work available on an Open Access basis (and intends to pay the relevant APC), the terms of reuse of such Open Access shall be governed by a Creative Commons licence – details of these licences and which Creative Commons licence will apply to this Work are set out in our licence referred to above.

Other than as permitted in any relevant BMJ Author's Self Archiving Policies, I confirm this Work has not been accepted for publication elsewhere, is not being considered for publication elsewhere and does not duplicate material already published. I confirm all authors consent to publication of this Work and authorise the granting of this licence.

For peer review only

Feasibility, usability, and acceptability of pediatric lung ultrasound among healthcare providers and caregivers for the diagnosis of childhood pneumonia in resource-constrained settings: A qualitative study

Authors: Atif Riaz[1], Olga Cambaco[2], Laura E Ellington[3]*, Jennifer L Lenahan[4], Khatia Munguambe[2], Usma Mehmood[1], Alessandro Lamorte[5], Sana Qaiser[1], Imran Nisar[1], Giovanni Volpicelli[6], Quique Bassat[7], Fyezah Jehan[1], Amy Sarah Ginsburg[8]

[1]Department of Pediatrics and Child Health, Aga Khan University, Karachi, Pakistan

[2]Centro de Investigação em Saúde de Manhiça (CISM), Maputo, Mozambique

[3]University of Washington, Seattle, WA, USA

[4]Save the Children, Seattle, WA, USA

[5]Department of Emergency Medicine, Parini Hospital, Aosta, Italy

[6]Department of Emergency Medicine, San Luigi Gonzaga University Hospital, Orbassano, Italy

[7]ISGlobal, Hospital Clínic - Universitat de Barcelona, Barcelona, Spain

[8]Clinical Trial Center, University of Washington, Seattle, WA, USA

*Corresponding author

E-mail: lelling@uw.edu

Address: 4800 Sand Point Way NE, Seattle, WA 98105

Word count: 4,810

For peer review only

ABSTRACT

Objectives: Pediatric pneumonia burden and mortality are highest in low- and middle-income countries (LMIC). Pediatric lung ultrasound (LUS) has emerged as a promising diagnostic tool for pneumonia in LMIC. Despite a growing evidence base for LUS use in pediatric pneumonia diagnosis, little is known about its potential for successful implementation in LMIC. Our objectives were to evaluate the feasibility, usability, and acceptability of LUS in the diagnosis of pediatric pneumonia.

Design: Prospective qualitative study using semi-structured interviews

Setting: Two referral hospitals in Mozambique and Pakistan

Participants: A total of 21 healthcare providers (HCPs) and 20 caregivers were enrolled.

Results: HCPs highlighted themes of limited resource availability for the feasibility of LUS implementation, including perceived high cost of equipment, maintenance demands, time constraints, and limited trained staff. HCPs emphasized the importance of policymaker support and caregiver acceptance for long-term success. HCP perspectives of usability highlighted ease of use and integration into existing workflow. HCPs and caregivers had positive attitudes towards LUS with few exceptions. Both HCPs and caregivers emphasized the potential for rapid, improved diagnosis of pediatric respiratory conditions using LUS.

Conclusions: This was the first study to evaluate HCP and caregiver perspectives of pediatric LUS through qualitative analysis. Critical components impacting feasibility, usability, and acceptability of LUS for pediatric pneumonia diagnosis in LMIC were identified for initial deployment. Future research should explore LUS sustainability, with a particular focus on quality control, device maintenance and functionality, and adoption of the new technology within the health system. This study highlights the need to engage both users and recipients of new technology early in order to adapt future interventions to the local context for successful implementation.

For peer review only

Trial registration: This study was registered NCT03187067 with ClinicalTrials.gov (https://clinicaltrials.gov/ct2/show/NCT03187067) on June 14, 2017.

For peer review only

Strengths and limitations of this study:

- This is the first study to evaluate important elements of successful LUS implementation through HCP and caregiver perspectives.
- We focused on themes of LUS feasibility, usability, and acceptability to help individuals and organizations to develop deployment strategies that focus on successful long-term sustainability.
- Our study was limited by a relatively small sample size at two study sites and may not be generalizable to other centers.
- We performed interviews prior to LUS implementation, requesting participants to reflect on their opinions with limited experience of LUS.

Keywords: Public health; paediatric thoracic medicine; qualitative research; ultrasonography; respiratory infections; international health services

For peer review only

INTRODUCTION

Pneumonia kills over 900,000 children under five years of age worldwide each year, the vast majority in low- and middle-income countries (LMIC).(1, 2) Pediatric pneumonia is challenging to diagnose, especially in settings where clinical expertise and diagnostic tools are not readily available or accessible. Chest radiography (CXR) is expensive, resource-intensive, carries ionizing radiation, and lacks sensitivity and specificity.(3-5) In LMIC, healthcare providers (HCPs) rely on nonspecific signs and symptoms, including cough, difficulty breathing, fast breathing, and chest indrawing, for the diagnosis and management of pneumonia based on the World Health Organization (WHO) guidelines for Integrated Management of Childhood Illness (IMCI).(6) Pediatric lung ultrasound (LUS) has emerged as a promising diagnostic tool for pneumonia in LMIC due to its diagnostic accuracy and reliability, portability, ease of use, lack of ionizing radiation, and lower cost compared to CXR.(7-15) Despite a growing evidence base for LUS use in pediatric pneumonia diagnosis, particularly in high-income settings, little is known about its potential for successful implementation in LMIC.

The research-to-practice gap is a well-described challenge, with evidence-based interventions taking an average of 17 years to be integrated into clinical practice, and even longer in LMIC.(16) This gap concedes that evidence alone for an intervention is not sufficient to ensure its successful uptake and sustainability in real-world settings. Implementation in LMIC carries additional challenges related to resource availability, clinical training, maintenance of devices, and time constraints. Successful implementation strategies suggest first assessing potential barriers and facilitators in order to adapt the intervention to the local context.(17) Therefore, the objective of this study was to evaluate the feasibility, usability, and acceptability of LUS through qualitative methods with HCPs and caregivers in Mozambique and Pakistan.

For peer review only - http://bmjopen.bmj.com/site/about/guidelines.xhtml

METHODS

We performed a qualitative evaluation as part of a larger pilot study evaluating whether adding LUS to WHO IMCI clinical assessment improves pneumonia diagnosis in young children. Briefly, 270 children aged two to 23 months presenting with cough and/or difficulty breathing were enrolled through routine screening at the two study sites in Mozambique and Pakistan. Children underwent LUS and CXR in addition to routine clinical assessment and were followed for a month after enrollment. Diagnostic accuracy of LUS was compared to CXR as well as to WHO IMCI clinical assessment alone. The complete protocol has been presented elsewhere.(18)

Setting

We recruited participants at two sites, Manhiça District Hospital (MDH) in Manhiça, Mozambique and Sindh Government Children's Hospital-Poverty Eradication Initiative (SGCH-PEI) in Karachi, Pakistan. Located 90 km from the capital city Maputo in a semi-rural district, MDH is a referral healthcare facility serving a population of 183,000. MDH has a 32-bed pediatric ward, 8-bed high-dependency unit, and 6-bed pediatric day hospital. SGCH-PEI is the district hospital for District Central, the largest district in Karachi, Pakistan. SGCH-PEI is equipped with more than 100 inpatient pediatric beds including a neonatal intensive care unit and has approximately 1000-1500 outpatient visits per day. CXR is available at both sites.

Participants

Participants were recruited from within the larger pilot study between April and May 2018 and included both HCPs and caregivers. Healthcare administrators and HCPs were eligible to participate if they 1) worked at one of the study sites, 2) were involved in or aware of the LUS study, and 3) had experience caring for children. HCPs were enrolled by convenience sampling, targeting a total of ten at each site to balance healthcare administration duties, experience with

For peer review only - http://bmjopen.bmj.com/site/about/guidelines.xhtml

LUS, and HCP role. Caregivers were eligible if they were at least 18 years of age, and had a child participating in the LUS study. Caregivers were approached sequentially about participating in this study during their child's enrollment in the larger pilot study until the target enrollment of ten caregivers at each site was reached.

Data collection

Researchers JLL and ASG developed the in-depth interview guides with review by AR and KM. Using the standardized interview guides and standard operating procedures to reduce inter-interviewer variability within and between sites, AR and KM, experienced in qualitative methods, supervised the on-site trainings of qualitative research assistants from the local community. Semi-structured interviews were conducted in person in the participant's preferred language by the trained research assistants using the standardized interview guide that did not change throughout data collection. Interviews lasted 30 to 45 minutes in duration and were performed in a private space within each healthcare setting. For HCPs and administrators, questions explored feasibility and acceptability of LUS, while HCPs were also asked specifically about usability of LUS. Feasibility questions explored challenges with healthcare delivery in its current state, experience with technology, storage of equipment, and additional perceptions of facilitators and barriers to integration of LUS within their healthcare facility. Usability questions targeted user's experience with LUS, particular difficulties with the device, and preferred level of HCPs to perform LUS. Acceptability questions focused on likes and dislikes regarding LUS, perceived value of LUS, and comparison to CXR. Questions for caregivers centered around acceptability of LUS in the care of their child, including caregivers' understanding of LUS, their likes and dislikes with the LUS exam, comparison to their experience with CXR for their child, and preference for availability of LUS in the future. All interviews were digitally recorded, transcribed with deidentified information, and translated into English. Transcriptions were reviewed and edited for grammar and clarity and therefore, were not considered direct quotes.

For peer review only - http://bmjopen.bmj.com/site/about/guidelines.xhtml

Data analysis

Participant characteristics were summarized using descriptive statistics (counts and proportions). Qualitative thematic analyses were performed by OC and KM in Mozambique, AR in Pakistan, and LEE using deductive coding. All had previous experience in qualitative coding and analysis. A deductive approach was used by manually coding transcripts using *a priori* topical codes chosen based on the primary research aims of this study: evaluation of feasibility, usability, and acceptability. We were less interested in developing new themes by an inductive approach and instead chose to employ an existing framework using themes describing implementation. *Feasibility* was defined as how easily HCPs/ administrators thought LUS could be integrated into the existing healthcare setting and included both individual and organizational attributes around perceived facilitators and barriers to using LUS within routine workflow. *Usability* was defined as the extent to which LUS operation was user-friendly in obtaining images and interpreting them. *Acceptability* was defined as the extent to which individuals performing (HCPs) or receiving (caregivers) the LUS exam considered it to be appropriate. These definitions were adapted from the implementation science literature.(19) Content analysis was supported by NVivo 11.0 software in Pakistan, while in Mozambique a matrix was manually developed using Microsoft Excel. Final factors and chosen quotes were reviewed by all authors across sites to ensure agreement of key findings. SRQR checklist for qualitative research was used to guide reporting of our methods and results.(20)

Ethics

The study was conducted in accordance with the International Conference on Harmonisation, Good Clinical Practice and the Declaration of Helsinki 2008, and was approved by the Western Institutional Review Board in the state of Washington, USA; the Comité Institucional de Bioética em Saúde do Centro de Investigação em Saúde de Manhiça (Manhiça, Mozambique); the Comité Nacional de Bioética em Saúde (Maputo, Mozambique, Ref. 246/CNBS/17); the Comité

For peer review only

For peer review only - http://bmjopen.bmj.com/site/about/guidelines.xhtml

de Ética del Hospital Clínic de Barcelona (Barcelona, Spain; ref. HCB/2017/0074) and the Aga Khan University Ethics Review Committee (Karachi, Pakistan). This study was registered NCT03187067 with ClinicalTrials.gov. All participants provided written informed consent in their preferred local language. Participant anonymity was maintained during data analysis and report writing.

Patient and public involvement

Patients were not involved in the design, or conduct, or reporting, or dissemination plans of this research.

RESULTS

Participant characteristics

A total of 21 HCPs and 20 caregivers were enrolled from both sites. Of the 21 HCPs, nine were physicians, three of whom had primarily administrative roles, four were nurses or a medical agent, two were radiology technicians, and six had other primary roles (Table 1). The LUS exams were performed by non-physician healthcare personnel at both sites, a nurse and a medical agent in Mozambique and two radiology technicians in Pakistan. Over half of the HCPs who participated in the study were female (55% in Pakistan and 60% in Mozambique). Eight HCPs were physicians in Pakistan (73%), while only one HCP was a physician in Mozambique (10%). Similarly, eight (73%) had graduated from university or higher in Pakistan, while two (20%) had in Mozambique. About half of the HCPs at both sites had more than 8 years of experience in their current role. Caregivers were all female and ranged in age from 19 to 42 years (Table 2). All but one caregiver was the mother of a child enrolled in the parent study, and all but one had a child aged less than twelve months enrolled. The majority of caregivers had completed primary (60%) or secondary (30%) school. Interviews with HCPs and caregivers

For peer review only - http://bmjopen.bmj.com/site/about/guidelines.xhtml

revealed major subthemes around feasibility, usability and acceptability of LUS implementation for diagnosing and managing childhood pneumonia (Table 3).

Table 1. Characteristics of healthcare provider participants.

	Mozambique N=10	Pakistan N=11
	N (%)	
**Female**	6 (60)	6 (55)
**Age (years)**		
20-35	4 (40)	3 (27)
36-60	6 (60)	8 (73)
**Provider role**		
Physician	1 (10)	8 (73)
Healthcare administrator[a]	0	3
Nurse or medical agent	4 (40)	0
Radiology technician	0	2 (18)
Research manager	1 (10)	0
Phlebotomist	0	1 (9)
Consent administrator	4 (40)	0
**Highest education achieved**		
Primary school	2 (20)	0
Secondary school	6 (60)	1 (9)
Technical school	0	2 (18)
University or higher	2 (20)	8 (73)
**Years of experience in current role**		
1-8	5 (50)	6 (55)
9-14	3 (30)	3 (27)
>14	2 (20)	2 (18)

[a] Healthcare administrators were also physicians.

Table 2. Characteristics of caregiver participants.

	Mozambique N=10	Pakistan N=10
	N (%)	
**Female**	10 (100)	10 (100)
**Age (years)**		
19-24	4 (40)	3 (30)
25-29	0	3 (30)
30-34	3 (30)	2 (20)
$\geq$ 35	3 (30)	2 (20)

For peer review only - http://bmjopen.bmj.com/site/about/guidelines.xhtml

**Age of child (months)**		
<12	10 (100)	9 (90)
12-23	0	1 (9)
**Relationship to child**		
Parent	9 (90)	9 (90)
Grandparent	1 (10)	0
Other	0	1 (10)
**Highest education achieved**		
Less than primary school	0	1 (10)
Primary school	9 (90)	3 (30)
Secondary school	1 (10)	4 (40)
University or higher	0	2 (20)

Table 3. Summary of major subthemes.

Domain	Subthemes	Explanation	Example questions from interview guide
**Feasibility**	Cost/resources	Includes cost and maintenance/storage of LUS equipment, sufficient number of adequately trained staff, potential increased workload and time	What should happen to make LUS successful?<br>Do you foresee any problems with keeping the LUS device in the hospital when not in use?<br>Do you think that LUS could be integrated into this facility? What would be some of the challenges/ barriers?<br>How much training (hours and/or days) do you think that learning to use LUS would take?
	Support from policymakers	Allocating sufficient resources to LUS equipment purchase and promoting use of LUS broadly across public hospital sector	
	Acceptance of caregivers	Buy-in from recipients of LUS	
**Usability**	Device management	Performing LUS image/video clip capture	Tell me about your experience with ultrasound.<br>What was easy and/or difficult to learn regarding LUS?<br>How much time did a LUS examination take?<br>What level of healthcare provider do you think should perform/ interpret LUS?
	Image interpretation	Making diagnosis using available LUS images/video clips	
	Integration into existing workflow	Using LUS as point-of-care tool within existing clinic structure	
**Acceptability**	Perceived value	Reasons respondent gave for liking and/or wanting LUS implemented within healthcare setting	What did you think about LUS? What do you like about LUS? What do you dislike?<br>Did you have any concerns about

For peer review only

	Affective attitude	Extent respondent liked or did not like LUS	LUS during your child's visit?<br>How do you think that the LUS would impact your child's care (caregivers) or ability to care for children (HCPs)?<br>How does your experience with LUS compare to CXR?
	Patient comfort	How child and caregiver tolerated LUS exam	
	General understanding	Respondent's ability to articulate indications and expectations of LUS	

LUS = lung ultrasound

For peer review only

Feasibility

HCPs identified three major factors affecting LUS feasibility: costs/resources; support from policymakers; and acceptance of caregivers. Identified costs associated with successful LUS implementation included equipment, appropriately trained staff, and time. About half of the HCPs interviewed in both Mozambique and Pakistan expressed concern with the cost of the LUS technology as a potential barrier for the widespread implementation of LUS as a diagnostic modality. Currently, ultrasound is not a readily available diagnostic tool in their settings and would require the purchase of a number of devices for use. HCPs in each site had different concerns about safe storage of the device. For example, one HCP in Pakistan and a majority in Mozambique were concerned about potential theft. A minority of HCPs, mostly in Mozambique, also expressed concern about maintenance of the LUS equipment over time.

...I bet it's not that affordable because if it was affordable, even the district hospital could have it since it is a large unit, and it would be something helpful to have.

HCP 812_0431, Mozambique

It is portable, small and delicate device so there are chances of theft. And since we are working in public sector hospital, so we have to be very careful for its protection.

HCP 6011, Pakistan

For peer review only - http://bmjopen.bmj.com/site/about/guidelines.xhtml

With poor maintenance, the quality of the functioning of the device will be compromised.
HCP 812_0362, Mozambique

In addition to the equipment itself, HCPs also commented on the importance of increasing and training staff to perform LUS. About half of the HCPs in both Mozambique and Pakistan quoted that successful LUS implementation relied on adequately trained staff to operate and interpret LUS.

Train more staff… to do LUS and interpret. I think the important thing is to train ... first, to train people. So, to be able to implement, then, the next phase, we will need serious lectures to help explain to the people… what is ultrasound, what is its purpose, and what the benefits of ultrasound are. Also, of course, one training session isn't enough because the hospital is very large.
HCP 812_0428, Mozambique

They have to increase the number of technicians, staff, and other requirements so that everything will be done in an organized way.
HCP 6004, Pakistan

Training for the pilot study consisted of a one-day standardized LUS training course as well as three days of closely supervised LUS practice at each site. Opinions on adequate length of training varied greatly across the two sites. In Pakistan, one HCP thought two to three days would suffice, while the remaining three who responded suggested at least two weeks would be required for adequate training. Notably, no participants mentioned refresher or repeat training for successful implementation.

For peer review only - http://bmjopen.bmj.com/site/about/guidelines.xhtml

Performing LUS may take time away from other provision of care in LMIC, as one HCP in Mozambique remarked:

> *Currently, when a CXR is ordered, the patient leaves while we are seeing other patients or in other activities. Then the patient comes back with the CXR. In the case of ultrasound, it has to be the clinician who is attending, doing the ultrasound, interpreting the ultrasound, and giving the result. So maybe it would take a little more time.*
> *HCP 812-0430, Mozambique*

Similarly, HCPs discussed the impact of LUS on time for the care of young children with respiratory illnesses. The majority of HCPs felt that LUS was fast and efficient due to its small size and portability. However, a few also felt that in a busy clinical setting, even if LUS is quick, it may be challenging to perform because of time restraints related to other essential duties.

A second important factor affecting feasibility of LUS was support from policymakers. This was brought up by HCPs with and without administrative roles in Pakistan. The majority of HCPs in Pakistan identified the importance of key stakeholder buy-in to promote policy change on a broader scale to improve implementation and sustainability of LUS in the public hospital sector.

> *An ultrasound and probe are so costly, and we have to purchase them. We would require documentation for purchase, and this process could be delayed. It depends on administration. They are the decision makers, and they can help us.*
> *HCP 6009, Pakistan*

For peer review only - http://bmjopen.bmj.com/site/about/guidelines.xhtml

Interestingly, this was not a theme that HCPs in Mozambique identified for successful LUS implementation; rather HCPs in Mozambique agreed that policymakers would gladly receive LUS, but only one HCP commented on the importance of policymakers for successful LUS implementation. Of note, no healthcare administrators were interviewed in Mozambique.

A third factor impacting feasibility of LUS was acceptance among caregivers. HCPs reported that for successful use, LUS must be accepted by caregivers through increased knowledge and practice. Most HCPs felt that caregivers would have high acceptability of LUS, but all highlighted the importance of caregiver buy-in for LUS to be successful. One HCP also mentioned the importance of acceptance by HCPs through rigorous research.

> *It is necessary to first explain to the children's mothers. Mothers still do not know and do not understand exactly what an ultrasound is, so it is necessary for the provider to explain to the mother in order to make them realize that this is a very important means of diagnosis. That, and that it doesn't hurt.*
> *HCP 812_0431, Mozambique*

> *Difficulties may be whether people are accepting it or not and what people think of it.*
> *HCP 6002, Pakistan*

> *Advocacy is best way to introduce [LUS], and dissemination of information is also a good way to create an awareness among people. First, you have to disseminate it to healthcare providers and give them confidence that it has good sensitivity, its diagnoses are correct, and you can save children from x-ray's radiations. If they are convinced, they promote it to the patients.*
> *HCP 6012, Pakistan*

For peer review only - http://bmjopen.bmj.com/site/about/guidelines.xhtml

Usability

HCPs with experience using the LUS device found the probe and interface easy to use, especially after practice, and that capturing LUS images was more straightforward than interpretation. Generally, HCPs felt that LUS was easiest to use and interpret in calm children without abnormal findings.

> *This application is very good. We performed it very well. We didn't face any difficulty in it. Everything is good in it. Sometimes little pathology in x-ray is not visible, but these are visible in this [LUS]… in my opinion it is good. This device is good. It is comfortable. Its probe is also good. So I didn't face any difficulty with it.*
>
> *HCP 6002, Pakistan*

> *As said, ultrasound is simple. It's simply a matter of capturing images… The most difficult thing is to understand the meaning of the images and to interpret. This is the most difficult part.*
>
> *HCP 812_0430, Mozambique*

Almost half of HCPs quoted challenges with high quality image capture when children were irritable or crying or if the identified pathology was small. A minority also reported difficulties with the LUS system, particularly with storing or losing images that were recently captured. Almost all HCPs agreed that HCPs who performed LUS could interpret the results.

> *Well, I think that who knows how to take the exam has to know how to interpret.*
>
> *HCP 812_0427, Mozambique*

For peer review only - http://bmjopen.bmj.com/site/about/guidelines.xhtml

HCPs at the two sites differed in their opinions regarding what level of HCPs should perform LUS exams. In Pakistan where two radiology technicians performed the LUS exams, HCPs generally agreed that radiologists or sonographers would be the most appropriate HCPs to perform this task compared to physicians, nurses, or other technicians who were evaluating the child. In Mozambique where a nurse and a medical agent performed the LUS exams, the majority of HCPs agreed that a nurse and/or physician with or without a technician could perform the LUS exam.

Interpretation can be done by a senior level person who must know all about ultrasound. Like, sonologists and physicians can do it.

HCP 6004, Pakistan

... for me, it does not have to be a doctor to perform it and know how to interpret it. So I think a health technician, a nurse, a medical agent can do it very well.

HCP 812_0427, Mozambique

Another aspect of LUS usability involved its ready integration within existing workflows. All HCPs agreed that LUS could be integrated within the existing workflow due to its small size, relatively quick exam, and portability. HCPs reported that LUS took about 15 to 20 minutes to complete.

This is portable device and easy to carry and handle…. The visibility of [the screen] is good and its quality is also good and a clear [video] clip can be saved with it.

HCP 6003, Pakistan

For peer review only - http://bmjopen.bmj.com/site/about/guidelines.xhtml

As a whole, I think it is easy to handle. It is accessible and easy to transport. You can leave it here, for example, to an area that is without electricity, and bring it to do the exam room and make a diagnosis. I think this is the most impressive of ultrasound.

HCP 812_0398, Mozambique

Acceptability

HCPs and caregivers reported four major factors affecting acceptability of the LUS device: perceived value of LUS; affective attitude; patient comfort; and general understanding of LUS. All HCPs and caregivers expressed that they liked LUS overall and described the perceived value of LUS, highlighting the rapid diagnostic ability of LUS, which included guiding definitive treatment during initial consultation instead of “guessing” the diagnosis.

Yes, it [should be incorporated into routine care], because as we have always had this problem of [bad] breathing in children. I think it would improve or help a lot. Now only those who enter the [research] study are lucky.

HCP 802_0443, Mozambique

I was satisfied because they do this to see the child's health, to know if the child is in good health or not, the child's breathing, here in the child's ribs and heart.

Caregiver 1098, Mozambique

It can be useful for doctor to immediately diagnose what has happened to child; otherwise they give medicine by guessing disease. I liked this ultrasound… It helps diagnose the disease quickly and it saves from wrong treatment due to accurate diagnosis.

For peer review only - http://bmjopen.bmj.com/site/about/guidelines.xhtml

Caregiver 3074, Pakistan

Caregivers were specifically asked if they would prefer bringing their child to a facility with LUS compared to one without, and all said yes.

HCPs also described benefits of LUS as a potential alternative compared to CXR with the primary and most common benefit reported being the lack of ionizing radiation with LUS. Just under half of the HCPs emphasized the additional benefit of streamlining workflow by using LUS as a point-of-care tool at the child's bedside, rather than having the child and family travel to the radiology department for a CXR. A few HCPs also added that CXR machines in their healthcare centers were not always functional due to lack of electricity or malfunctioning equipment. All but one caregiver also reported that they liked LUS as much or more than CXR.

We get an instant diagnosis without radiation.

HCP 6004, Pakistan

It could change the dynamics of the unit itself. Because suppose there is a huge queue of patients who want to have an X-ray. They will stay in the queue for a long time. Now, with the pulmonary ultrasound, if there are two, three devices, there is an examiner over there, another one over there and ready, the child entered, examined, and left. That would streamline this in a way.

HCP 812_0427, Mozambique

While the vast majority of comments regarding LUS were positive, a few caregivers from Pakistan commented that their children were agitated during the LUS exam. Two caregivers

For peer review only - http://bmjopen.bmj.com/site/about/guidelines.xhtml

were worried that their children's agitation was an indication that LUS was harmful to their children. No caregivers from Mozambique reported concerns with LUS.

Ultrasound takes more time [than CXR], and the child also gets irritated.

Caregiver 3094, Pakistan

I was worried that my child should not get hurt. I was afraid a little bit.

Caregiver 3077, Pakistan

When asked about their understanding of LUS, all HCPs were able to correctly identify the indication for LUS. A minority of caregivers were still unclear about the purpose of LUS after the exam was explained and performed by study staff. Some HCPs and caregivers had higher expectations of LUS than what was explained to them.

It shows clear picture of inside body part of child tells about all hidden diseases.

Caregiver 3075, Pakistan

I like it because the probe makes it easier for me to diagnose problems, such as pneumonia and other pathologies that I can see when I have an ultrasound scan.

HCP 812_0431, Mozambique

DISCUSSION

There is a growing body of literature supporting the use of LUS for the diagnosis of pediatric pneumonia across many healthcare settings. However, few studies have looked at feasibility of LUS deployment in LMIC, and none have explored perspectives of those using or receiving the

For peer review only

intervention: HCPs and caregivers. This study therefore adds to the implementation science literature of new technologies in LMIC for improved diagnosis and management of acute respiratory illness in young children. This was the first study to evaluate HCP and caregiver perspectives of pediatric LUS through qualitative analysis, specifically their thoughts on feasibility, usability, and acceptability of LUS in their healthcare setting. Themes associated with LUS feasibility from the HCP perspective included concerns of working in a resource-constrained environment, namely cost of equipment, limited trained staff, and time constraints, as well as the importance of policymaker support and caregiver acceptance. Regarding LUS usability, HCPs highlighted ease of image capture compared to interpretation, challenge of performing LUS in small, crying children, appropriate experience level of HCP, and integration into existing workflow. LUS acceptability themes from both HCPs and caregivers emphasized perceived value of LUS, such as rapid diagnosis, no ionizing radiation compared to CXR, and streamlining workflow, as well as the importance of caregiver/HCP understanding of LUS. Among both HCP and caregivers, there was an overall positive attitude with few exceptions.

Quality control

In this study, HCPs had varying opinions regarding length of a LUS training program, from a few days to multiple weeks. While high quality initial training is critically important to ensure successful roll-out of an intervention, perhaps more important is developing a system for quality monitoring over time with opportunities for refresher training. For example, as part of our pilot study, we monitored quality of LUS acquisition and interpretation in real-time. One of the study sites had lower quality images identified early in data collection. In response to this, we performed a series of remote refresher trainings via video conferencing. We tailored this intervention to the needs of the end user, first again reviewing basic use of the LUS probe for high quality image capture and followed up with a session on using the LUS probe in different ways to investigate pathology. Quality control is also important to address the high turnover of

For peer review only - http://bmjopen.bmj.com/site/about/guidelines.xhtml

HCPs and the overwhelming shortage of highly skilled HCPs that require task-shifting to less specialized HCPs.(21-24) These shortages underscore the need for ongoing quality control monitoring and efficient, high quality, and readily available training. Fortunately, there are different options to address these challenges in LMIC, including development of local experts to train the trainers, growing opportunities for remote learning through video conferencing, and reducing the need for training by incorporating artificial intelligence into LUS image acquisition and interpretation.

Device maintenance and functionality

While the importance of maintenance and equipment functionality was not a major theme identified by the HCPs or administrators in this study, these are important factors to consider in the adoption of LUS (or any new technology). The ultrasound system used in this study consisted of three parts: a probe, a removable cord, and a third-party tablet with an application as the collection interface. Various parts of this system failed at different points during the study and required replacement parts shipped from the United States. To make LUS adoption feasible long-term, the ultrasound selected should have a local supplier able to provide maintenance and replacements, as well as technical documentation and support in the local language. Secondly, prior to scale-up, budgets for devices and projections for device maintenance should be clearly defined by organizations providing and receiving the intervention. Thirdly, provisions for back-up parts and devices should be explored with an understanding of who is responsible for repairing the device and what is an acceptable turn-around time for repair.

Our findings were similar to prior qualitative research on the implementation of point-of-care ultrasound and more broadly, point-of-care diagnostics in LMIC.(25-27) Key stakeholder perspectives revealed that training as well as cost and maintenance of materials were major barriers to use, while improved diagnostic accuracy, timely diagnosis, and portability were major

For peer review only - http://bmjopen.bmj.com/site/about/guidelines.xhtml

benefits.(25-27) When considering point-of-care diagnostics more broadly, stakeholders emphasized cost-effectiveness evaluations and improving quality management systems, again supporting the importance of cost containment and quality of healthcare delivery through trained staff and functional equipment for sustainability.(26)

Acceptance and adoption of new technology

Quality control and device functionality ensure that the building blocks for successful implementation are in place and can be sustained over time. However, these building blocks are meaningless without the buy-in from healthcare leadership and frontline providers. Our study supports high acceptability of LUS by both HCPs and caregivers. While acceptability is an important first step, it is not sufficient to ensure high uptake by health systems. The Consolidated Framework for Implementation Research (CFIR) highlights the importance of evaluating the inner setting (e.g., structural characteristics, culture, and teamwork) and outer setting (e.g., external policies and incentives) to determine successful implementation.(28) This means that the setting in which the intervention is applied is almost as important as the intervention itself. For LUS to be successful, engagement by policymakers to develop guidelines, policy statements, and incentives will encourage systematic uptake by health centers. Furthermore, working with HCPs to integrate LUS into existing workflows within their busy clinical setting should attempt to streamline patient care without adding to the already high burden of care.

Limitations

There were several limitations to this study. While we performed training with interviewers prior to data collection to increase consistency across sites, the differences between sites may have contributed to interviewers asking questions differently, leading to differing responses between sites. Although we interviewed a wide range of participants with different experiences, it is

For peer review only - http://bmjopen.bmj.com/site/about/guidelines.xhtml

possible that we missed important perceptions limited by our sample size. We were unable to recruit healthcare administrators in Mozambique. Therefore, HCP responses in Mozambique reflected the opinions of frontline HCPs and not stakeholders in healthcare administration as was the case in Pakistan. We also recruited HCPs with varying experience with LUS, some of whom were involved in the larger pilot study. Participation by some interviewees in the LUS pilot study may have contributed to response bias, with HCPs responding more favorably to LUS and neglecting to discuss their negative opinions. Additionally, although transcripts were transcribed and translated, it is possible that some important concepts were missed or misinterpreted in the analysis process.

Importantly, HCPs discussed themes that were most relevant to initial deployment of LUS in their healthcare setting. This study was performed as part of a pilot study prior to implementation in their healthcare facility. HCP experience was therefore, limited to brief exposure to the device without experiencing first-hand challenges with sustainability. Future directions should include understanding barriers and facilitators to sustainability in LMIC following real-world experience with a particular focus on quality control, device maintenance and functionality, and acceptance and adoption of the new technology.

Conclusions

Through qualitative analysis, we identified several important components impacting feasibility, usability, and acceptability of LUS for the diagnosis of pediatric pneumonia in LMIC. HCPs and caregivers liked LUS for its perceived rapid results and the potential for improved diagnostic accuracy of pneumonia at bedside, lack of ionizing radiation, and potential for improved clinic workflow. HCPs thought it could be successfully integrated into their healthcare setting with sufficient training, knowledge sharing, policymaker buy-in, and caregiver acceptance. Potential barriers included cost and maintenance of LUS equipment, adequately trained staff, and comfort

For peer review only - http://bmjopen.bmj.com/site/about/guidelines.xhtml

level of both HCPs and caregivers with a new technology. Taken together, this study highlights the importance of early engagement of both users and recipients of new technology in order to adapt future interventions to the local context for successful implementation.

For peer review only

For peer review only - http://bmjopen.bmj.com/site/about/guidelines.xhtml

ACKNOWLEDGEMENTS

We thank the dedicated study staff at Manhiça District Hospital in Manhiça, Mozambique and Sindh Government Children's Hospital–Poverty Eradication Initiative in Karachi, Pakistan for implementing the study and providing patient care. We thank Cayetana Verastegui and Carla Pinto in Manhiça, Mozambique for their contributions to the study. We also thank the trial participants, their caregivers, and the local community in Manhiça, Mozambique and Karachi, Pakistan for their participation and support. CISM is supported by the Government of Mozambique and the Spanish Agency for International Development. ISGlobal receives support from the Spanish Ministry of Science and Innovation through the "Centro de Excelencia Severo Ochoa 2019-2023" Program (CEX2018-000806-S), and support from the Generalitat de Catalunya through the CERCA Program.

Author contributions: JLL and ASG designed the study and data collection instruments with input from AR, KM, AL, IN, GV, QB, and FJ. AL and GV provided lung ultrasound training and support. JLL and ASG coordinated and supervised data collection from the sites and IN, FJ, and QB supervised their respective sites. AR, OC, KM, UM, SQ, and IN acquired and managed the data. AR, OC, and KM analyzed and interpreted the data and produced study reports. LEE analyzed and interpreted the data using information from the study reports and wrote the manuscript with critical input from JLL and ASG. All authors worked collaboratively to review and approve the final manuscript.

Competing interests: None declared

Funding: This work was supported by grants from the Bill and Melinda Gates Foundation (OPP1105080) and Save the Children.

Data sharing: Data will be made available upon reasonable request to the corresponding author.

For peer review only

REFERENCES

1. Liu L, Oza S, Hogan D, Perin J, Rudan I, Lawn JE, et al. Global, regional, and national causes of child mortality in 2000–13, with projections to inform post-2015 priorities: an updated systematic analysis. The Lancet. 2015;385(9966):430-40.

2. World Health Organization. Pneumonia: Fact Sheet 2019 [Available from: https://www.who.int/en/news-room/fact-sheets/detail/pneumonia.

3. Hopstaken RM, Witbraad T, van Engelshoven JM, Dinant GJ. Inter-observer variation in the interpretation of chest radiographs for pneumonia in community-acquired lower respiratory tract infections. Clin Radiol. 2004;59(8):743-52.

4. Williams GJ, Macaskill P, Kerr M, Fitzgerald DA, Isaacs D, Codarini M, et al. Variability and accuracy in interpretation of consolidation on chest radiography for diagnosing pneumonia in children under 5 years of age. Pediatr Pulmonol. 2013;48(12):1195-200.

5. Mazrani W, McHugh K, Marsden PJ. The radiation burden of radiological investigations. Arch Dis Child. 2007;92(12):1127-31.

6. World Health Organization. Integrated Management of Childhood Illness 2014.

7. Pereda MA, Chavez MA, Hooper-Miele CC, Gilman RH, Steinhoff MC, Ellington LE, et al. Lung ultrasound for the diagnosis of pneumonia in children: a meta-analysis. Pediatrics. 2015;135(4):714-22.

8. Chavez MA, Naithani N, Gilman RH, Tielsch JM, Khatry S, Ellington LE, et al. Agreement Between the World Health Organization Algorithm and Lung Consolidation Identified Using Point-of-Care Ultrasound for the Diagnosis of Childhood Pneumonia by General Practitioners. Lung. 2015;193(4):531-8.

9. Ellington LE, Gilman RH, Chavez MA, Pervaiz F, Marin-Concha J, Compen-Chang P, et al. Lung ultrasound as a diagnostic tool for radiographically-confirmed pneumonia in low resource settings. Respir Med. 2017;128:57-64.

For peer review only - http://bmjopen.bmj.com/site/about/guidelines.xhtml

10. Cortellaro F, Colombo S, Coen D, Duca PG. Lung ultrasound is an accurate diagnostic tool for the diagnosis of pneumonia in the emergency department. Emerg Med J. 2012;29(1):19-23.

11. Reissig A, Copetti R, Mathis G, Mempel C, Schuler A, Zechner P, et al. Lung ultrasound in the diagnosis and follow-up of community-acquired pneumonia: a prospective, multicenter, diagnostic accuracy study. Chest. 2012;142(4):965-72.

12. Ho MC, Ker CR, Hsu JH, Wu JR, Dai ZK, Chen IC. Usefulness of lung ultrasound in the diagnosis of community-acquired pneumonia in children. Pediatr Neonatol. 2015;56(1):40-5.

13. Berce V, Tomazin M, Gorenjak M, Berce T, Lovrencic B. The Usefulness of Lung Ultrasound for the Aetiological Diagnosis of Community-Acquired Pneumonia in Children. Sci Rep. 2019;9(1):17957.

14. Jones BP, Tay ET, Elikashvili I, Sanders JE, Paul AZ, Nelson BP, et al. Feasibility and Safety of Substituting Lung Ultrasonography for Chest Radiography When Diagnosing Pneumonia in Children: A Randomized Controlled Trial. Chest. 2016;150(1):131-8.

15. Brogi E, Bignami E, Sidoti A, Shawar M, Gargani L, Vetrugno L, et al. Could the use of bedside lung ultrasound reduce the number of chest x-rays in the intensive care unit? Cardiovasc Ultrasound. 2017;15(1):23.

16. Glasgow RE, Vinson C, Chambers DK, M. J., Kaplan RM, Hunter C. National Institutes of Health Approaches to Dissemination and Implementation Science: Current and Future Directions. Am J Pub Health. 2012;102(7):1274-81.

17. Graham ID, Logan J, Harrison MB, Straus SE, Tetroe J, Caswell W, et al. Lost in knowledge translation: time for a map? J Contin Educ Health Prof. 2006;26(1):13-24.

18. Lenahan JL, Volpicelli G, Lamorte A, Jehan F, Bassat Q, Ginsburg AS. Multicentre pilot study evaluation of lung ultrasound for the management of paediatric pneumonia in low-resource settings: a study protocol. BMJ Open Respir Res. 2018;5(1):e000340.

For peer review only

19. Lewis CC, Fischer S, Weiner BJ, Stanick C, Kim M, Martinez RG. Outcomes for implementation science: an enhanced systematic review of instruments using evidence-based rating criteria. Implement Sci. 2015;10:155.

20. O'Brien BC, Harris IB, Beckman TJ, Reed DA, Cook DA. Standards for reporting qualitative research: a synthesis of recommendations. Acad Med. 2014;89(9):1245-51.

21. Aluttis C, Bishaw T, Frank MW. The workforce for health in a globalized context--global shortages and international migration. Global health action. 2014;7:23611.

22. Puchalski Ritchie LM, Khan S, Moore JE, Timmings C, van Lettow M, Vogel JP, et al. Low- and middle-income countries face many common barriers to implementation of maternal health evidence products. J Clin Epidemiol. 2016;76:229-37.

23. Liu JX, Goryakin Y, Maeda A, Bruckner T, Scheffler R. Global Health Workforce Labor Market Projections for 2030. Hum Resour Health. 2017;15(1):11.

24. Seidman G, Atun R. Does task shifting yield cost savings and improve efficiency for health systems? A systematic review of evidence from low-income and middle-income countries. Hum Resour Health. 2017;15(1):29.

25. Maw AM, Galvin B, Henri R, Yao M, Exame B, Fleshner M, et al. Stakeholder Perceptions of Point-of-Care Ultrasound Implementation in Resource-Limited Settings. Diagnostics (Basel). 2019;9(4).

26. Mashamba-Thompson TP, Jama NA, Sartorius B, Drain PK, Thompson RM. Implementation of Point-of-Care Diagnostics in Rural Primary Healthcare Clinics in South Africa: Perspectives of Key Stakeholders. Diagnostics (Basel). 2017;7(1).

27. Drain PK, Hyle EP, Noubary F, Freedberg KA, Wilson D, Bishai WR, et al. Diagnostic point-of-care tests in resource-limited settings. The Lancet Infectious Diseases. 2014;14(3):239-49.

For peer review only - http://bmjopen.bmj.com/site/about/guidelines.xhtml

28. Damschroder LJ, Aron DC, Keith RE, Kirsh SR, Alexander JA, Lowery JC. Fostering implementation of health services research findings into practice: a consolidated framework for advancing implementation science. Implement Sci. 2009;4:50.

For peer review only

For peer review only - http://bmjopen.bmj.com/site/about/guidelines.xhtml

Reporting checklist for qualitative study.

Based on the SRQR guidelines.

Instructions to authors

Complete this checklist by entering the page numbers from your manuscript where readers will find each of the items listed below.

Your article may not currently address all the items on the checklist. Please modify your text to include the missing information. If you are certain that an item does not apply, please write "n/a" and provide a short explanation.

Upload your completed checklist as an extra file when you submit to a journal.

In your methods section, say that you used the SRQRreporting guidelines, and cite them as:

O'Brien BC, Harris IB, Beckman TJ, Reed DA, Cook DA. Standards for reporting qualitative research: a synthesis of recommendations. Acad Med. 2014;89(9):1245-1251.

		Reporting Item	Page Number
**Title**			
	#1	Concise description of the nature and topic of the study identifying the study as qualitative or indicating the approach (e.g. ethnography, grounded theory) or data collection methods (e.g. interview, focus group) is recommended	1
**Abstract**			
	#2	Summary of the key elements of the study using the abstract format of the intended publication; typically includes background, purpose, methods, results and conclusions	2
**Introduction**			
Problem formulation	#3	Description and signifcance of the problem / phenomenon studied: review of relevant theory and empirical work; problem statement	5
Purpose or research question	#4	Purpose of the study and specific objectives or questions	5

For peer review only

Methods

Qualitative approach and research paradigm	#5	Qualitative approach (e.g. ethnography, grounded theory, case study, phenomenolgy, narrative research) and guiding theory if appropriate; identifying the research paradigm (e.g. postpositivist, constructivist / interpretivist) is also recommended; rationale. The rationale should briefly discuss the justification for choosing that theory, approach, method or technique rather than other options available; the assumptions and limitations implicit in those choices and how those choices influence study conclusions and transferability. As appropriate the rationale for several items might be discussed together.	8
Researcher characteristics and reflexivity	#6	Researchers' characteristics that may influence the research, including personal attributes, qualifications / experience, relationship with participants, assumptions and / or presuppositions; potential or actual interaction between researchers' characteristics and the research questions, approach, methods, results and / or transferability	7
Context	#7	Setting / site and salient contextual factors; rationale	6
Sampling strategy	#8	How and why research participants, documents, or events were selected; criteria for deciding when no further sampling was necessary (e.g. sampling saturation); rationale	6-7
Ethical issues pertaining to human subjects	#9	Documentation of approval by an appropriate ethics review board and participant consent, or explanation for lack thereof; other confidentiality and data security issues	8-9
Data collection methods	#10	Types of data collected; details of data collection procedures including (as appropriate) start and stop dates of data collection and analysis, iterative process, triangulation of sources / methods, and modification of procedures in response to evolving study findings; rationale	7
Data collection instruments and technologies	#11	Description of instruments (e.g. interview guides, questionnaires) and devices (e.g. audio recorders) used for data collection; if / how the instruments(s) changed over the course of the study	7

For peer review only

Units of study	#12	Number and relevant characteristics of participants, documents, or events included in the study; level of participation (could be reported in results)	9-10
Data processing	#13	Methods for processing data prior to and during analysis, including transcription, data entry, data management and security, verification of data integrity, data coding, and anonymisation / deidentification of excerpts	7,8
Data analysis	#14	Process by which inferences, themes, etc. were identified and developed, including the researchers involved in data analysis; usually references a specific paradigm or approach; rationale	8
Techniques to enhance trustworthiness	#15	Techniques to enhance trustworthiness and credibility of data analysis (e.g. member checking, audit trail, triangulation); rationale	8
**Results/findings**			
Syntheses and interpretation	#16	Main findings (e.g. interpretations, inferences, and themes); might include development of a theory or model, or integration with prior research or theory	12-20
Links to empirical data	#17	Evidence (e.g. quotes, field notes, text excerpts, photographs) to substantiate analytic findings	12-20
**Discussion**			
Intergration with prior work, implications, transferability and contribution(s) to the field	#18	Short summary of main findings; explanation of how findings and conclusions connect to, support, elaborate on, or challenge conclusions of earlier scholarship; discussion of scope of application / generalizability; identification of unique contributions(s) to scholarship in a discipline or field	20-23
Limitations	#19	Trustworthiness and limitations of findings	23-24
**Other**			
Conflicts of interest	#20	Potential sources of influence of perceived influence on study conduct and conclusions; how these were managed	25
Funding	#21	Sources of funding and other support; role of funders in data collection, interpretation and reporting	25

For peer review only

None The SRQR checklist is distributed with permission of Wolters Kluwer © 2014 by the Association of American Medical Colleges. This checklist can be completed online using https://www.goodreports.org/, a tool made by the EQUATOR Network in collaboration with Penelope.ai

For peer review only

BMJ Open

Feasibility, usability, and acceptability of pediatric lung ultrasound among healthcare providers and caregivers for the diagnosis of childhood pneumonia in resource-constrained settings: A qualitative study

Journal:	*BMJ Open*
Manuscript ID	bmjopen-2020-042547.R1
Article Type:	Original research
Date Submitted by the Author:	30-Jan-2021

Complete List of Authors:	Riaz, Atif; Aga Khan University, Pediatrics and Child Health<br>Cambaco, Olga; Centro de Investigação em Saúde de Manhiça<br>Ellington, Laura; University of Washington School of Medicine, Pediatrics<br>Lenahan, Jennifer; Save the Children Federation Inc<br>Munguambe, Khatia ; Centro de Investigacao em Saude de Manhica<br>Mehmood, Usma; Aga Khan University, Pediatrics and Child Health<br>Lamorte, Alessandro; Umberto Parini Hospital, Emergency Medicine<br>Qaisar, Sana; Aga Khan University, Pediatrics and Child Health<br>Baloch, Benazir; The Aga Khan University, Pediatrics and Child Health<br>Kanth, Neel; Sindh Government Hospital Karachi, Children's Hospital-Poverty Eradication Initiative<br>Nisar, Muhammad; Aga Khan University, Pediatrics and Child Health<br>Volpicelli, Giovanni; San Luigi Gonzaga University Hospital, Emergency Medicine<br>Bassat, Quique; ISGLOBAL; Universitat de Barcelona<br>Jehan, Fyezah; Aga Khan University, Paediatrics and Child Health<br>ginsburg, amy sarah; University of Washington, Clinical Trial Center

**Primary Subject Heading**:	Global health
Secondary Subject Heading:	Paediatrics, Public health, Qualitative research, Respiratory medicine, Radiology and imaging
Keywords:	Paediatric thoracic medicine < PAEDIATRICS, PUBLIC HEALTH, QUALITATIVE RESEARCH, ULTRASONOGRAPHY, Respiratory infections < THORACIC MEDICINE, International health services < HEALTH SERVICES ADMINISTRATION & MANAGEMENT

SCHOLARONE™
Manuscripts

I, the Submitting Author has the right to grant and does grant on behalf of all authors of the Work (as defined in the below author licence), an exclusive licence and/or a non-exclusive licence for contributions from authors who are: i) UK Crown employees; ii) where BMJ has agreed a CC-BY licence shall apply, and/or iii) in accordance with the terms applicable for US Federal Government officers or employees acting as part of their official duties; on a worldwide, perpetual, irrevocable, royalty-free basis to BMJ Publishing Group Ltd ("BMJ") its licensees and where the relevant Journal is co-owned by BMJ to the co-owners of the Journal, to publish the Work in this journal and any other BMJ products and to exploit all rights, as set out in our licence.

The Submitting Author accepts and understands that any supply made under these terms is made by BMJ to the Submitting Author unless you are acting as an employee on behalf of your employer or a postgraduate student of an affiliated institution which is paying any applicable article publishing charge ("APC") for Open Access articles. Where the Submitting Author wishes to make the Work available on an Open Access basis (and intends to pay the relevant APC), the terms of reuse of such Open Access shall be governed by a Creative Commons licence – details of these licences and which Creative Commons licence will apply to this Work are set out in our licence referred to above.

Other than as permitted in any relevant BMJ Author's Self Archiving Policies, I confirm this Work has not been accepted for publication elsewhere, is not being considered for publication elsewhere and does not duplicate material already published. I confirm all authors consent to publication of this Work and authorise the granting of this licence.

For peer review only

Feasibility, usability, and acceptability of pediatric lung ultrasound among healthcare providers and caregivers for the diagnosis of childhood pneumonia in resource-constrained settings: A qualitative study

Authors: Atif Riaz[1], Olga Cambaco[2], Laura E Ellington[3]*, Jennifer L Lenahan[4], Khatia Munguambe[2], Usma Mehmood[1], Alessandro Lamorte[5], Sana Qaisar[1], Benazir Baloch[1], Neel Kanth[6], Muhammad Imran Nisar[1], Giovanni Volpicelli[7], Quique Bassat[8], Fyezah Jehan[1], Amy Sarah Ginsburg[9]

[1]Department of Pediatrics and Child Health, Aga Khan University, Karachi, Pakistan

[2]Centro de Investigação em Saúde de Manhiça (CISM), Maputo, Mozambique

[3]University of Washington, Seattle, WA, USA

[4]Save the Children, Seattle, WA, USA

[5]Department of Emergency Medicine, Parini Hospital, Aosta, Italy

[6]Sindh Government Children's Hospital-Poverty Eradication Initiative, Karachi, Pakistan

[7]Department of Emergency Medicine, San Luigi Gonzaga University Hospital, Orbassano, Italy

[8]ISGlobal, Hospital Clínic - Universitat de Barcelona, Barcelona, Spain

[9]Clinical Trial Center, University of Washington, Seattle, WA, USA

*Corresponding author: Laura E. Ellington, 4800 Sand Point Way NE, Seattle, WA 98105; lelling@uw.edu

Word count: 4,884

For peer review only

ABSTRACT

Objectives: Pediatric pneumonia burden and mortality are highest in low- and middle-income countries (LMIC). Pediatric lung ultrasound (LUS) has emerged as a promising diagnostic tool for pneumonia in LMIC. Despite a growing evidence base for LUS use in pediatric pneumonia diagnosis, little is known about its potential for successful implementation in LMIC. Our objectives were to evaluate the feasibility, usability, and acceptability of LUS in the diagnosis of pediatric pneumonia.

Design: Prospective qualitative study using semi-structured interviews

Setting: Two referral hospitals in Mozambique and Pakistan

Participants: A total of 21 healthcare providers (HCPs) and 20 caregivers were enrolled.

Results: HCPs highlighted themes of limited resource availability for the feasibility of LUS implementation, including perceived high cost of equipment, maintenance demands, time constraints, and limited trained staff. HCPs emphasized the importance of policymaker support and caregiver acceptance for long-term success. HCP perspectives of usability highlighted ease of use and integration into existing workflow. HCPs and caregivers had positive attitudes towards LUS with few exceptions. Both HCPs and caregivers emphasized the potential for rapid, improved diagnosis of pediatric respiratory conditions using LUS.

Conclusions: This was the first study to evaluate HCP and caregiver perspectives of pediatric LUS through qualitative analysis. Critical components impacting feasibility, usability, and acceptability of LUS for pediatric pneumonia diagnosis in LMIC were identified for initial deployment. Future research should explore LUS sustainability, with a particular focus on quality control, device maintenance and functionality, and adoption of the new technology within the health system. This study highlights the need to engage both users and recipients of new technology early in order to adapt future interventions to the local context for successful implementation.

For peer review only - http://bmjopen.bmj.com/site/about/guidelines.xhtml

Trial registration: This study was registered NCT03187067 with ClinicalTrials.gov (https://clinicaltrials.gov/ct2/show/NCT03187067) on June 14, 2017.

For peer review only

For peer review only - http://bmjopen.bmj.com/site/about/guidelines.xhtml

Strengths and limitations of this study:

- This is the first study to evaluate important elements of successful LUS implementation through HCP and caregiver perspectives.
- We focused on themes of LUS feasibility, usability, and acceptability to help individuals and organizations to develop deployment strategies that focus on successful long-term sustainability.
- We performed interviews prior to LUS implementation, requesting participants to reflect on their opinions with limited experience of LUS.

Keywords: Public health; paediatric thoracic medicine; qualitative research; ultrasonography; respiratory infections; international health services

For peer review only

INTRODUCTION

Pneumonia kills over 900,000 children under five years of age worldwide each year, the vast majority in low- and middle-income countries (LMIC).(1, 2) Pediatric pneumonia is challenging to diagnose, especially in settings where clinical expertise and diagnostic tools are not readily available or accessible. Chest radiography (CXR) is expensive, resource-intensive, carries ionizing radiation, and lacks sensitivity and specificity.(3-5) In LMIC, healthcare providers (HCPs) rely on nonspecific signs and symptoms, including cough, difficulty breathing, fast breathing, and chest indrawing, for the diagnosis and management of pneumonia based on the World Health Organization (WHO) guidelines for Integrated Management of Childhood Illness (IMCI).(6) Pediatric lung ultrasound (LUS) has emerged as a promising diagnostic tool for pneumonia in LMIC due to its diagnostic accuracy and reliability, portability, ease of use, lack of ionizing radiation, and lower cost compared to CXR.(7-15) Despite a growing evidence base for LUS use in pediatric pneumonia diagnosis, particularly in high-income settings, little is known about its potential for successful implementation in LMIC.

The research-to-practice gap is a well-described challenge, with evidence-based interventions taking an average of 17 years to be integrated into clinical practice, and even longer in LMIC.(16) This gap concedes that evidence alone for an intervention is not sufficient to ensure its successful uptake and sustainability in real-world settings. Implementation in LMIC carries additional challenges related to resource availability, clinical training, maintenance of devices, and time constraints.(17-19) Successful implementation strategies suggest first assessing potential barriers and facilitators in order to adapt the intervention to the local context.(20) Therefore, the objective of this study was to evaluate the feasibility, usability, and acceptability of LUS through qualitative methods with HCPs and caregivers in Mozambique and Pakistan.

For peer review only - http://bmjopen.bmj.com/site/about/guidelines.xhtml

METHODS

We performed a qualitative evaluation as part of a larger pilot study evaluating whether adding LUS to WHO IMCI clinical assessment improves pneumonia diagnosis in young children. Briefly, 270 children aged two to 23 months presenting with cough and/or difficulty breathing were enrolled through routine screening at the two study sites in Mozambique and Pakistan. Children underwent LUS and CXR in addition to routine clinical assessment and were followed for a month after enrollment. Diagnostic accuracy of LUS was compared to CXR as well as to WHO IMCI clinical assessment alone. The complete protocol has been presented elsewhere.(21)

Setting

We recruited participants at two sites, Manhiça District Hospital (MDH) in Manhiça, Mozambique and Sindh Government Children's Hospital-Poverty Eradication Initiative (SGCH-PEI) in Karachi, Pakistan. Located 90 km from the capital city Maputo in a semi-rural district, MDH is a referral healthcare facility serving a population of 183,000. MDH has a 32-bed pediatric ward, 8-bed high-dependency unit, and 6-bed pediatric day hospital, and is typically staffed with at least 2 pediatricians, 6 general physicians, and larger numbers of nurses and medical agents. Under-five mortality rate in Mozambique is estimated to be 87.2/1000 live births with 19% of post-neonatal deaths (aged 1-59 months) caused by pneumonia.(22) SGCH-PEI is the district hospital for District Central, the largest district in Karachi, Pakistan. SGCH-PEI is equipped with more than 100 inpatient pediatric beds including a neonatal intensive care unit and has approximately 1000-1500 pediatric outpatient visits per day. SGCH-PEI is staffed with 4 pediatricians and a larger number of nurses. Under-five mortality rate in Pakistan is estimated to be 85.5/1000 live births with pneumonia the number one cause of post-neonatal deaths, at 29%.(23) Neither site has access to sub-specialty pediatric consultation. Digital CXR is available at both sites.

For peer review only - http://bmjopen.bmj.com/site/about/guidelines.xhtml

Participants

Participants were recruited from within the larger pilot study between April and May 2018 and included both HCPs and caregivers. Healthcare administrators and HCPs were eligible to participate if all of the following criteria were met: 1) employed at one of the study sites; 2) involved in or aware of the LUS study; and 3) had experience caring for children. HCPs were enrolled by convenience sampling, targeting a total of ten at each site to balance healthcare administration duties, experience with LUS, and HCP role. Caregivers were eligible if they were at least 18 years of age and were a primary caregiver for a child enrolled in the study. Caregivers were approached sequentially about participating in this study during their child's enrollment in the larger pilot study until the target enrollment of ten caregivers at each site was reached.

Data collection

Researchers JLL and ASG developed the in-depth interview guides with review by AR and KM. Using the standardized interview guides and standard operating procedures to reduce inter-interviewer variability within and between sites, AR and KM, experienced in qualitative methods, supervised the on-site trainings of qualitative research assistants from the local community. Semi-structured interviews were conducted in person in the participant's preferred language by the trained research assistants using the standardized interview guide that did not change throughout data collection (Supplement S1). Interviews lasted 30 to 45 minutes in duration and were performed in a private space within each healthcare setting. For HCPs and administrators, questions explored feasibility and acceptability of LUS, while HCPs were also asked specifically about usability of LUS. Feasibility questions explored challenges with healthcare delivery in its current state, experience with technology, storage of equipment, and additional perceptions of facilitators and barriers to integration of LUS within their healthcare facility. Usability questions targeted user's experience with LUS, particular difficulties with the device, and preferred level of

For peer review only - http://bmjopen.bmj.com/site/about/guidelines.xhtml

HCPs to perform LUS. Acceptability questions focused on likes and dislikes regarding LUS, perceived value of LUS, and comparison to CXR. Questions for caregivers centered around acceptability of LUS in the care of their child, including caregivers' understanding of LUS, their likes and dislikes with the LUS exam, comparison to their experience with CXR for their child, and preference for availability of LUS in the future. All interviews were digitally recorded, transcribed with deidentified information, and translated into English. Transcriptions were reviewed and edited for grammar and clarity and therefore, were not considered direct quotes.

Data analysis

Participant characteristics were summarized using descriptive statistics (counts and proportions). Qualitative thematic analyses were performed by OC and KM in Mozambique, AR in Pakistan, and LEE using deductive coding. All had previous experience in qualitative coding and analysis. A deductive approach was used by manually coding transcripts using *a priori* topical codes chosen based on the primary research aims of this study: evaluation of feasibility, usability, and acceptability. We were less interested in developing new themes by an inductive approach and instead chose to employ an existing framework using themes describing implementation. *Feasibility* was defined as how easily HCPs/ administrators thought LUS could be integrated into the existing healthcare setting and included both individual and organizational attributes around perceived facilitators and barriers to using LUS within routine workflow. *Usability* was defined as the extent to which LUS operation was user-friendly in obtaining images and interpreting them. *Acceptability* was defined as the extent to which individuals performing (HCPs) or receiving (caregivers) the LUS exam considered it to be appropriate. These definitions were adapted from the implementation science literature.(24) Data analysis was supported by NVivo 11.0 software in Pakistan, while in Mozambique a matrix was manually developed using Microsoft Excel. Final factors and chosen quotes were reviewed by all authors

For peer review only

For peer review only - http://bmjopen.bmj.com/site/about/guidelines.xhtml

across sites to ensure agreement of key findings. SRQR checklist for qualitative research was used to guide reporting of our methods and results.(25)

Ethics

The study was conducted in accordance with the International Conference on Harmonisation, Good Clinical Practice and the Declaration of Helsinki 2008, and was approved by the Western Institutional Review Board in the state of Washington, USA; the Comité Institucional de Bioética em Saúde do Centro de Investigação em Saúde de Manhiça (Manhiça, Mozambique, Ref. 084/2017); the Comité Nacional de Bioética em Saúde (Maputo, Mozambique, Ref. 246/CNBS/17); the Comité de Ética del Hospital Clínic de Barcelona (Barcelona, Spain; Ref. HCB/2017/0074) and the Aga Khan University Ethics Review Committee (Karachi, Pakistan). This study was registered NCT03187067 with ClinicalTrials.gov. All participants provided written informed consent in their preferred local language. Participant anonymity was maintained during data analysis and report writing.

Patient and public involvement

Patients were not involved in the design, or conduct, or reporting, or dissemination plans of this research.

RESULTS

Participant characteristics

A total of 21 HCPs and 20 caregivers were enrolled from both sites. Of the 21 HCPs, nine were physicians, three of whom had primarily administrative roles, four were nurses or a medical agent, two were radiology technicians, and six had other primary roles (Table 1). The LUS exams were performed by non-physician healthcare personnel at both sites, a nurse and a medical agent in Mozambique and two radiology technicians in Pakistan. Over half of the HCPs who participated in the study were female (55% in Pakistan and 60% in Mozambique). Eight

For peer review only

HCPs were physicians in Pakistan (73%), while only one HCP was a physician in Mozambique (10%). Similarly, eight (73%) had graduated from university or higher in Pakistan, while two (20%) had in Mozambique. About half of the HCPs at both sites had more than 8 years of experience in their current role. Caregivers were all female and ranged in age from 19 to 42 years (Table 2). All but one caregiver was the mother of a child enrolled in the parent study, and all but one had a child aged less than twelve months enrolled. The majority of caregivers had completed primary (60%) or secondary (30%) school. Interviews with HCPs and caregivers revealed major subthemes around feasibility, usability and acceptability of LUS implementation for diagnosing and managing childhood pneumonia (Table 3).

Table 1. Characteristics of healthcare provider participants.

	Mozambique N=10	Pakistan N=11
	N (%)	
**Female**	6 (60)	6 (55)
**Age (years)**		
20-35	4 (40)	3 (27)
36-60	6 (60)	8 (73)
**Provider role**		
Physician	1 (10)	8 (73)
Healthcare administrator[a]	0	3
Nurse or medical agent	4 (40)	0
Radiology technician	0	2 (18)
Research manager	1 (10)	0
Phlebotomist	0	1 (9)
Consent administrator	4 (40)	0
**Highest education achieved**		
Primary school	2 (20)	0
Secondary school	6 (60)	1 (9)
Technical school	0	2 (18)
University or higher	2 (20)	8 (73)
**Years of experience in current role**		
1-8	5 (50)	6 (55)
9-14	3 (30)	3 (27)
>14	2 (20)	2 (18)

[a] Healthcare administrators were also physicians.

For peer review only - http://bmjopen.bmj.com/site/about/guidelines.xhtml

Table 2. Characteristics of caregiver participants.

	Mozambique N=10	Pakistan N=10
	N (%)	
**Female**	10 (100)	10 (100)
**Age (years)**		
19-24	4 (40)	3 (30)
25-29	0	3 (30)
30-34	3 (30)	2 (20)
$\geq$ 35	3 (30)	2 (20)
**Age of child (months)**		
<12	10 (100)	9 (90)
12-23	0	1 (9)
**Relationship to child**		
Parent	9 (90)	9 (90)
Grandparent	1 (10)	0
Other	0	1 (10)
**Highest education achieved**		
Less than primary school	0	1 (10)
Primary school	9 (90)	3 (30)
Secondary school	1 (10)	4 (40)
University or higher	0	2 (20)

Table 3. Summary of major subthemes.

Domain	Subthemes	Explanation	Example questions from interview guide
**Feasibility**	Cost/resources	Includes cost and maintenance/storage of LUS equipment, sufficient number of adequately trained staff, potential increased workload and time	What should happen to make LUS successful?<br>Do you foresee any problems with keeping the LUS device in the hospital when not in use?<br>Do you think that LUS could be integrated into this facility? What would be some of the challenges/ barriers?<br>How much training (hours and/or days) do you think that learning to use LUS would take?
	Support from policymakers	Allocating sufficient resources to LUS equipment purchase and promoting use of LUS broadly across public hospital sector	
	Acceptance of caregivers	Buy-in from recipients of LUS	
**Usability**	Device management	Performing LUS image/video clip capture	Tell me about your experience with ultrasound.

For peer review only - http://bmjopen.bmj.com/site/about/guidelines.xhtml

	Image interpretation	Making diagnosis using available LUS images/video clips	What was easy and/or difficult to learn regarding LUS?<br>How much time did a LUS examination take?<br>What level of healthcare provider do you think should perform/ interpret LUS?
	Integration into existing workflow	Using LUS as point-of-care tool within existing clinic structure	
**Acceptability**	Perceived value	Reasons respondent gave for liking and/or wanting LUS implemented within healthcare setting	What did you think about LUS? What do you like about LUS? What do you dislike?<br>Did you have any concerns about LUS during your child's visit?<br>How do you think that the LUS would impact your child's care (caregivers) or ability to care for children (HCPs)?<br>How does your experience with LUS compare to CXR?
	Affective attitude	Extent respondent liked or did not like LUS	
	Patient comfort	How child and caregiver tolerated LUS exam	
	General understanding	Respondent's ability to articulate indications and expectations of LUS	

LUS = lung ultrasound

Feasibility

HCPs identified three major factors affecting LUS feasibility: costs/resources; support from policymakers; and acceptance of caregivers. Identified costs associated with successful LUS implementation included equipment, appropriately trained staff, and time. About half of the HCPs interviewed in both Mozambique and Pakistan expressed concern with the cost of the LUS technology as a potential barrier for the widespread implementation of LUS as a diagnostic modality. Currently, ultrasound is not a readily available diagnostic tool in their settings and would require the purchase of a number of devices for use. HCPs in each site had different concerns about safe storage of the device. For example, one HCP in Pakistan and a majority in Mozambique were concerned about potential theft. A minority of HCPs, mostly in Mozambique, also expressed concern about maintenance of the LUS equipment over time.

For peer review only

For peer review only - http://bmjopen.bmj.com/site/about/guidelines.xhtml

...I bet it's not that affordable because if it was affordable, even the district hospital could have it since it is a large unit, and it would be something helpful to have.

HCP, Mozambique

It is portable, small and delicate device so there are chances of theft. And since we are working in public sector hospital, so we have to be very careful for its protection.

HCP, Pakistan

With poor maintenance, the quality of the functioning of the device will be compromised.

HCP, Mozambique

In addition to the equipment itself, HCPs also commented on the importance of recruiting and training staff to perform LUS. About half of the HCPs in both Mozambique and Pakistan quoted that successful LUS implementation relied on adequately trained staff to operate and interpret LUS.

Train more staff… to do LUS and interpret. I think the important thing is to train ... first, to train people. So, to be able to implement, then, the next phase, we will need serious lectures to help explain to the people… what is ultrasound, what is its purpose, and what the benefits of ultrasound are. Also, of course, one training session isn't enough because the hospital is very large.

HCP, Mozambique

They have to increase the number of technicians, staff, and other requirements so that everything will be done in an organized way.

HCP, Pakistan

For peer review only - http://bmjopen.bmj.com/site/about/guidelines.xhtml

Training for the pilot study consisted of a one-day standardized LUS training course as well as three days of closely supervised LUS practice at each site. Opinions on adequate length of training varied greatly across the two sites. In Pakistan, one HCP thought two to three days would suffice, while the remaining three who responded suggested at least two weeks would be required for adequate training. Notably, no participants mentioned refresher or repeat training for successful implementation.

Performing LUS may take time away from other provision of care in LMIC, as one HCP in Mozambique remarked:

> *Currently, when a CXR is ordered, the patient leaves while we are seeing other patients or in other activities. Then the patient comes back with the CXR. In the case of ultrasound, it has to be the clinician who is attending, doing the ultrasound, interpreting the ultrasound, and giving the result. So maybe it would take a little more time.*
> *HCP, Mozambique*

Similarly, HCPs discussed the impact of LUS on time for the care of young children with respiratory illnesses. The majority of HCPs felt that LUS was fast and efficient due to its small size and portability. However, a few also felt that in a busy clinical setting, even if LUS is quick, it may be challenging to perform because of time restraints related to other essential duties.

A second important factor affecting feasibility of LUS was support from policymakers. This was brought up by HCPs with and without administrative roles in Pakistan. The majority of HCPs in Pakistan identified the importance of key stakeholder buy-in to promote policy change on a broader scale to improve implementation and sustainability of LUS in the public hospital sector.

For peer review only - http://bmjopen.bmj.com/site/about/guidelines.xhtml

An ultrasound and probe are so costly, and we have to purchase them. We would require documentation for purchase, and this process could be delayed. It depends on administration. They are the decision makers, and they can help us.

HCP, Pakistan

Interestingly, this was not a theme that HCPs in Mozambique identified for successful LUS implementation; rather HCPs in Mozambique agreed that policymakers would gladly receive LUS, but only one HCP commented on the importance of policymakers for successful LUS implementation. Of note, no healthcare administrators were interviewed in Mozambique.

A third factor impacting feasibility of LUS was acceptance among caregivers. HCPs reported that for successful use, LUS must be accepted by caregivers through increased knowledge and practice. Most HCPs felt that caregivers would have high acceptability of LUS, but all highlighted the importance of caregiver buy-in for LUS to be successful. One HCP also mentioned the importance of acceptance by HCPs through rigorous research.

It is necessary to first explain to the children's mothers. Mothers still do not know and do not understand exactly what an ultrasound is, so it is necessary for the provider to explain to the mother in order to make them realize that this is a very important means of diagnosis. That, and that it doesn't hurt.

HCP, Mozambique

Difficulties may be whether people are accepting it or not and what people think of it.

HCP, Pakistan

For peer review only - http://bmjopen.bmj.com/site/about/guidelines.xhtml

Advocacy is best way to introduce [LUS], and dissemination of information is also a good way to create an awareness among people. First, you have to disseminate it to healthcare providers and give them confidence that it has good sensitivity, its diagnoses are correct, and you can save children from x-ray's radiations. If they are convinced, they promote it to the patients.

HCP, Pakistan

Usability

HCPs with experience using the LUS device found the probe and interface easy to use, especially after practice, and that capturing LUS images was more straightforward than interpretation. Generally, HCPs felt that LUS was easiest to use and interpret in calm children without abnormal findings.

This application is very good. We performed it very well. We didn't face any difficulty in it. Everything is good in it. Sometimes little pathology in x-ray is not visible, but these are visible in this [LUS]… in my opinion it is good. This device is good. It is comfortable. Its probe is also good. So I didn't face any difficulty with it.

HCP, Pakistan

As said, ultrasound is simple. It's simply a matter of capturing images… The most difficult thing is to understand the meaning of the images and to interpret. This is the most difficult part.

HCP, Mozambique

For peer review only

Almost half of HCPs quoted challenges with high quality image capture when children were irritable or crying or if the identified pathology was small. A minority also reported difficulties with the LUS system, particularly with storing or losing images that were recently captured. Almost all HCPs agreed that HCPs who performed LUS could interpret the results.

> *Well, I think that who knows how to take the exam has to know how to interpret.*
> *HCP, Mozambique*

HCPs at the two sites differed in their opinions regarding what level of HCPs should perform LUS exams. In Pakistan where two radiology technicians performed the LUS exams, HCPs generally agreed that radiologists or sonographers would be the most appropriate HCPs to perform this task compared to physicians, nurses, or other technicians who were evaluating the child. In Mozambique where a nurse and a medical agent performed the LUS exams, the majority of HCPs agreed that a nurse and/or physician with or without a technician could perform the LUS exam.

> *Interpretation can be done by a senior level person who must know all about ultrasound. Like, sonologists and physicians can do it.*
> *HCP, Pakistan*

> *... for me, it does not have to be a doctor to perform it and know how to interpret it. So I think a health technician, a nurse, a medical agent can do it very well.*
> *HCP, Mozambique*

Another aspect of LUS usability involved its ready integration within existing workflows. All HCPs agreed that LUS could be integrated within the existing workflow due to its small size,

For peer review only - http://bmjopen.bmj.com/site/about/guidelines.xhtml

For peer review only

relatively quick exam, and portability. HCPs reported that LUS took about 15 to 20 minutes to complete.

> *This is portable device and easy to carry and handle…. The visibility of [the screen] is good and its quality is also good and a clear [video] clip can be saved with it.*
> *HCP, Pakistan*

> *As a whole, I think it is easy to handle. It is accessible and easy to transport. You can leave it here, for example, to an area that is without electricity, and bring it to do the exam room and make a diagnosis. I think this is the most impressive of ultrasound.*
> *HCP, Mozambique*

Acceptability

HCPs and caregivers reported four major factors affecting acceptability of the LUS device: perceived value of LUS; affective attitude; patient comfort; and general understanding of LUS. All HCPs and caregivers expressed that they liked LUS overall and described the perceived value of LUS, highlighting the rapid diagnostic ability of LUS, which included guiding definitive treatment during initial consultation instead of "guessing" the diagnosis.

> *Yes, it [should be incorporated into routine care], because as we have always had this problem of [bad] breathing in children. I think it would improve or help a lot. Now only those who enter the [research] study are lucky.*
> *HCP, Mozambique*

For peer review only

I was satisfied because they do this to see the child's health, to know if the child is in good health or not, the child's breathing, here in the child's ribs and heart.

Caregiver, Mozambique

It can be useful for doctor to immediately diagnose what has happened to child; otherwise they give medicine by guessing disease. I liked this ultrasound… It helps diagnose the disease quickly and it saves from wrong treatment due to accurate diagnosis.

Caregiver, Pakistan

Caregivers were specifically asked if they would prefer bringing their child to a facility with LUS compared to one without, and all said yes.

HCPs also described benefits of LUS as a potential alternative compared to CXR with the primary and most common benefit reported being the lack of ionizing radiation with LUS. Just under half of the HCPs emphasized the additional benefit of streamlining workflow by using LUS as a point-of-care tool at the child's bedside, rather than having the child and family travel to the radiology department for a CXR. A few HCPs also added that CXR machines in their healthcare centers were not always functional due to lack of electricity or malfunctioning equipment. All but one caregiver also reported that they liked LUS as much or more than CXR.

We get an instant diagnosis without radiation.

HCP, Pakistan

It could change the dynamics of the unit itself. Because suppose there is a huge queue of patients who want to have an X-ray. They will stay in the queue for a long time. Now,

For peer review only - http://bmjopen.bmj.com/site/about/guidelines.xhtml

with the pulmonary ultrasound, if there are two, three devices, there is an examiner over there, another one over there and ready, the child entered, examined, and left. That would streamline this in a way.

HCP, Mozambique

While the vast majority of comments regarding LUS were positive, a few caregivers from Pakistan commented that their children were agitated during the LUS exam. Two caregivers were worried that their children's agitation was an indication that LUS was harmful to their children. No caregivers from Mozambique reported concerns with LUS.

Ultrasound takes more time [than CXR], and the child also gets irritated.

Caregiver, Pakistan

I was worried that my child should not get hurt. I was afraid a little bit.

Caregiver, Pakistan

When asked about their understanding of LUS, all HCPs were able to correctly identify the indication for LUS. A minority of caregivers were still unclear about the purpose of LUS after the exam was explained and performed by study staff. Some HCPs and caregivers had higher expectations of LUS than what was explained to them.

It shows clear picture of inside body part of child tells about all hidden diseases.

Caregiver, Pakistan

I like it because the probe makes it easier for me to diagnose problems, such as pneumonia and other pathologies that I can see when I have an ultrasound scan.

For peer review only - http://bmjopen.bmj.com/site/about/guidelines.xhtml

For peer review only

HCP, Mozambique

DISCUSSION

There is a growing body of literature supporting the use of LUS for the diagnosis of pediatric pneumonia across many healthcare settings. However, few studies have looked at feasibility of LUS deployment in LMIC, and none have explored perspectives of those using or receiving the intervention: HCPs and caregivers. This study therefore adds to the implementation science literature of new technologies in LMIC for improved diagnosis and management of acute respiratory illness in young children. This was the first study to evaluate HCP and caregiver perspectives of pediatric LUS through qualitative analysis, specifically their thoughts on feasibility, usability, and acceptability of LUS in their healthcare setting. Themes associated with LUS feasibility from the HCP perspective included concerns of working in a resource-constrained environment, namely cost of equipment, limited trained staff, and time constraints, as well as the importance of policymaker support and caregiver acceptance. Regarding LUS usability, HCPs highlighted ease of image capture compared to interpretation, challenge of performing LUS in small, crying children, appropriate experience level of HCP, and integration into existing workflow. LUS acceptability themes from both HCPs and caregivers emphasized perceived value of LUS, such as rapid diagnosis, no ionizing radiation compared to CXR, and streamlining workflow, as well as the importance of caregiver/HCP understanding of LUS. Among both HCP and caregivers, there was an overall positive attitude with few exceptions.

Quality control

In this study, HCPs had varying opinions regarding length of a LUS training program, from a few days to multiple weeks. While high quality initial training is critically important to ensure successful roll-out of an intervention, perhaps more important is developing a system for quality monitoring over time with opportunities for refresher training. For example, as part of our pilot

For peer review only - http://bmjopen.bmj.com/site/about/guidelines.xhtml

study, we monitored quality of LUS acquisition and interpretation in real-time. One of the study sites had lower quality images identified early in data collection. In response to this, we performed a series of remote refresher trainings via video conferencing. We tailored this intervention to the needs of the end user, first again reviewing basic use of the LUS probe for high quality image capture and followed up with a session on using the LUS probe in different ways to investigate pathology. Quality control is also important to address the high turnover of HCPs and the overwhelming shortage of highly skilled HCPs that require task-shifting to less specialized HCPs.(26-29) These shortages underscore the need for ongoing quality control monitoring and efficient, high quality, and readily available training. Fortunately, there are different options to address these challenges in LMIC, including development of local experts to train the trainers, growing opportunities for remote learning through video conferencing, and reducing the need for training by incorporating artificial intelligence into LUS image acquisition and interpretation.

Device maintenance and functionality

While the importance of maintenance and equipment functionality was not a major theme identified by the HCPs or administrators in this study, these are important factors to consider in the adoption of LUS (or any new technology). The ultrasound system used in this study consisted of three parts: a probe, a removable cord, and a third-party tablet with an application as the collection interface. Various parts of this system failed at different points during the study and required replacement parts shipped from the United States. To make LUS adoption feasible long-term, the ultrasound selected should have a local supplier able to provide maintenance and replacements, as well as technical documentation and support in the local language. Secondly, prior to scale-up, budgets for devices and projections for device maintenance should be clearly defined by organizations providing and receiving the intervention. Thirdly, provisions for back-up

For peer review only

parts and devices should be explored with an understanding of who is responsible for repairing the device and what is an acceptable turn-around time for repair.

Our findings were similar to prior qualitative research on the implementation of point-of-care ultrasound and more broadly, point-of-care diagnostics in LMIC.(30-32) Key stakeholder perspectives revealed that training as well as cost and maintenance of materials were major barriers to use, while improved diagnostic accuracy, timely diagnosis, and portability were major benefits.(30-32) When considering point-of-care diagnostics more broadly, stakeholders emphasized cost-effectiveness evaluations and improving quality management systems, again supporting the importance of cost containment and quality of healthcare delivery through trained staff and functional equipment for sustainability.(31)

Acceptance and adoption of new technology

Quality control and device functionality ensure that the building blocks for successful implementation are in place and can be sustained over time. However, these building blocks are meaningless without the buy-in from healthcare leadership and frontline providers. Our study supports high acceptability of LUS by both HCPs and caregivers. While acceptability is an important first step, it is not sufficient to ensure high uptake by health systems. The Consolidated Framework for Implementation Research (CFIR) highlights the importance of evaluating the inner setting (e.g., structural characteristics, culture, and teamwork) and outer setting (e.g., external policies and incentives) to determine successful implementation.(33) This means that the setting in which the intervention is applied is almost as important as the intervention itself. For LUS to be successful, engagement by policymakers to develop guidelines, policy statements, and incentives will encourage systematic uptake by health centers. Furthermore, working with HCPs to integrate LUS into existing workflows within their

For peer review only - http://bmjopen.bmj.com/site/about/guidelines.xhtml

busy clinical setting should attempt to streamline patient care without adding to the already high burden of care.

Limitations

There were several limitations to this study. While we performed training with interviewers prior to data collection to increase consistency across sites, the differences between sites may have contributed to interviewers asking questions differently, leading to differing responses between sites. Although we interviewed a wide range of participants with different experiences, it is possible that we missed important perceptions limited by our sample size. We were unable to recruit healthcare administrators in Mozambique. Therefore, HCP responses in Mozambique reflected the opinions of frontline HCPs and not stakeholders in healthcare administration as was the case in Pakistan. We also recruited HCPs with varying experience with LUS, some of whom were involved in the larger pilot study. Participation by some interviewees in the LUS pilot study may have contributed to response bias, with HCPs responding more favorably to LUS and neglecting to discuss their negative opinions. Additionally, although transcripts were transcribed and translated, it is possible that some important concepts were missed or misinterpreted in the analysis process.

Importantly, HCPs discussed themes that were most relevant to initial deployment of LUS in their healthcare setting. This study was performed as part of a pilot study prior to implementation in their healthcare facility. HCP experience was therefore, limited to brief exposure to the device without experiencing first-hand challenges with sustainability. Future directions should include understanding barriers and facilitators to sustainability in LMIC following real-world experience with a particular focus on quality control, device maintenance and functionality, and acceptance and adoption of the new technology.

For peer review only - http://bmjopen.bmj.com/site/about/guidelines.xhtml

Conclusions

Through qualitative analysis, we identified several important components impacting feasibility, usability, and acceptability of LUS for the diagnosis of pediatric pneumonia in LMIC. HCPs and caregivers liked LUS for its perceived rapid results and the potential for improved diagnostic accuracy of pneumonia at bedside, lack of ionizing radiation, and potential for improved clinic workflow. HCPs thought it could be successfully integrated into their healthcare setting with sufficient training, knowledge sharing, policymaker buy-in, and caregiver acceptance. Potential barriers included cost and maintenance of LUS equipment, adequately trained staff, and comfort level of both HCPs and caregivers with a new technology. Taken together, this study highlights the importance of early engagement of both users and recipients of new technology in order to adapt future interventions to the local context for successful implementation.

For peer review only

ACKNOWLEDGEMENTS

We thank the dedicated study staff at Manhiça District Hospital in Manhiça, Mozambique and Sindh Government Children's Hospital–Poverty Eradication Initiative in Karachi, Pakistan for implementing the study and providing patient care. We thank Cayetana Verastegui and Carla Pinto in Manhiça, Mozambique for their contributions to the study. We also thank the trial participants, their caregivers, and the local community in Manhiça, Mozambique and Karachi, Pakistan for their participation and support. CISM is supported by the Government of Mozambique and the Spanish Agency for International Development. ISGlobal receives support from the Spanish Ministry of Science and Innovation through the "Centro de Excelencia Severo Ochoa 2019-2023" Program (CEX2018-000806-S), and support from the Generalitat de Catalunya through the CERCA Program.

Author contributions: JLL and ASG designed the study and data collection instruments with input from AR, KM, AL, MIN, GV, QB, and FJ. AL and GV provided lung ultrasound training and support. JLL and ASG coordinated and supervised data collection from the sites and NK, MIN, FJ, and QB supervised their respective sites. AR, OC, KM, UM, SQ, BB, and MIN acquired and managed the data. AR, OC, and KM analyzed and interpreted the data and produced study reports. LEE analyzed and interpreted the data using information from the study reports and wrote the manuscript with critical input from JLL and ASG. All authors worked collaboratively to review and approve the final manuscript.

Competing interests: None declared

Funding: This work was supported by grants from the Bill and Melinda Gates Foundation (OPP1105080) and Save the Children.

Data sharing: Data will be made available upon reasonable request to the corresponding author.

For peer review only

REFERENCES

1. Liu L, Oza S, Hogan D, Perin J, Rudan I, Lawn JE, et al. Global, regional, and national causes of child mortality in 2000–13, with projections to inform post-2015 priorities: an updated systematic analysis. The Lancet. 2015;385(9966):430-40.

2. World Health Organization. Pneumonia: Fact Sheet 2019 [Available from: https://www.who.int/en/news-room/fact-sheets/detail/pneumonia.

3. Hopstaken RM, Witbraad T, van Engelshoven JM, Dinant GJ. Inter-observer variation in the interpretation of chest radiographs for pneumonia in community-acquired lower respiratory tract infections. Clin Radiol. 2004;59(8):743-52.

4. Williams GJ, Macaskill P, Kerr M, Fitzgerald DA, Isaacs D, Codarini M, et al. Variability and accuracy in interpretation of consolidation on chest radiography for diagnosing pneumonia in children under 5 years of age. Pediatr Pulmonol. 2013;48(12):1195-200.

5. Mazrani W, McHugh K, Marsden PJ. The radiation burden of radiological investigations. Arch Dis Child. 2007;92(12):1127-31.

6. World Health Organization. Integrated Management of Childhood Illness 2014.

7. Pereda MA, Chavez MA, Hooper-Miele CC, Gilman RH, Steinhoff MC, Ellington LE, et al. Lung ultrasound for the diagnosis of pneumonia in children: a meta-analysis. Pediatrics. 2015;135(4):714-22.

8. Chavez MA, Naithani N, Gilman RH, Tielsch JM, Khatry S, Ellington LE, et al. Agreement Between the World Health Organization Algorithm and Lung Consolidation Identified Using Point-of-Care Ultrasound for the Diagnosis of Childhood Pneumonia by General Practitioners. Lung. 2015;193(4):531-8.

9. Ellington LE, Gilman RH, Chavez MA, Pervaiz F, Marin-Concha J, Compen-Chang P, et al. Lung ultrasound as a diagnostic tool for radiographically-confirmed pneumonia in low resource settings. Respir Med. 2017;128:57-64.

For peer review only - http://bmjopen.bmj.com/site/about/guidelines.xhtml

10. Cortellaro F, Colombo S, Coen D, Duca PG. Lung ultrasound is an accurate diagnostic tool for the diagnosis of pneumonia in the emergency department. Emerg Med J. 2012;29(1):19-23.

11. Reissig A, Copetti R, Mathis G, Mempel C, Schuler A, Zechner P, et al. Lung ultrasound in the diagnosis and follow-up of community-acquired pneumonia: a prospective, multicenter, diagnostic accuracy study. Chest. 2012;142(4):965-72.

12. Ho MC, Ker CR, Hsu JH, Wu JR, Dai ZK, Chen IC. Usefulness of lung ultrasound in the diagnosis of community-acquired pneumonia in children. Pediatr Neonatol. 2015;56(1):40-5.

13. Berce V, Tomazin M, Gorenjak M, Berce T, Lovrencic B. The Usefulness of Lung Ultrasound for the Aetiological Diagnosis of Community-Acquired Pneumonia in Children. Sci Rep. 2019;9(1):17957.

14. Jones BP, Tay ET, Elikashvili I, Sanders JE, Paul AZ, Nelson BP, et al. Feasibility and Safety of Substituting Lung Ultrasonography for Chest Radiography When Diagnosing Pneumonia in Children: A Randomized Controlled Trial. Chest. 2016;150(1):131-8.

15. Brogi E, Bignami E, Sidoti A, Shawar M, Gargani L, Vetrugno L, et al. Could the use of bedside lung ultrasound reduce the number of chest x-rays in the intensive care unit? Cardiovasc Ultrasound. 2017;15(1):23.

16. Glasgow RE, Vinson C, Chambers DK, M. J., Kaplan RM, Hunter C. National Institutes of Health Approaches to Dissemination and Implementation Science: Current and Future Directions. Am J Pub Health. 2012;102(7):1274-81.

17. Sanders D, Haines A. Implementation research is needed to achieve international health goals. PLoS Med. 2006;3(6):e186.

18. Madon T, Hofman KJ, Kupfer L, Glass RI. Public health. Implementation science. Science. 2007;318(5857):1728-9.

For peer review only

19. Means AR, Kemp CG, Gwayi-Chore MC, Gimbel S, Soi C, Sherr K, et al. Evaluating and optimizing the consolidated framework for implementation research (CFIR) for use in low- and middle-income countries: a systematic review. Implement Sci. 2020;15(1):17.

20. Graham ID, Logan J, Harrison MB, Straus SE, Tetroe J, Caswell W, et al. Lost in knowledge translation: time for a map? J Contin Educ Health Prof. 2006;26(1):13-24.

21. Lenahan JL, Volpicelli G, Lamorte A, Jehan F, Bassat Q, Ginsburg AS. Multicentre pilot study evaluation of lung ultrasound for the management of paediatric pneumonia in low-resource settings: a study protocol. BMJ Open Respir Res. 2018;5(1):e000340.

22. Neonatal and Child Country Profile: Mozambique: World Health Organization; 2016 [Available from: http://www.who.int/maternal_child_adolescent/epidemiology/profiles/neonatal_child/moz.pdf.

23. Neonatal and Child Country Profile: Pakistan: World Health Organization 2016 [Available from: http://www.who.int/maternal_child_adolescent/epidemiology/profiles/neonatal_child/pak.pdf?ua=1.

24. Lewis CC, Fischer S, Weiner BJ, Stanick C, Kim M, Martinez RG. Outcomes for implementation science: an enhanced systematic review of instruments using evidence-based rating criteria. Implement Sci. 2015;10:155.

25. O'Brien BC, Harris IB, Beckman TJ, Reed DA, Cook DA. Standards for reporting qualitative research: a synthesis of recommendations. Acad Med. 2014;89(9):1245-51.

26. Aluttis C, Bishaw T, Frank MW. The workforce for health in a globalized context--global shortages and international migration. Global health action. 2014;7:23611.

27. Puchalski Ritchie LM, Khan S, Moore JE, Timmings C, van Lettow M, Vogel JP, et al. Low- and middle-income countries face many common barriers to implementation of maternal health evidence products. J Clin Epidemiol. 2016;76:229-37.

For peer review only

28. Liu JX, Goryakin Y, Maeda A, Bruckner T, Scheffler R. Global Health Workforce Labor Market Projections for 2030. Hum Resour Health. 2017;15(1):11.

29. Seidman G, Atun R. Does task shifting yield cost savings and improve efficiency for health systems? A systematic review of evidence from low-income and middle-income countries. Hum Resour Health. 2017;15(1):29.

30. Maw AM, Galvin B, Henri R, Yao M, Exame B, Fleshner M, et al. Stakeholder Perceptions of Point-of-Care Ultrasound Implementation in Resource-Limited Settings. Diagnostics (Basel). 2019;9(4).

31. Mashamba-Thompson TP, Jama NA, Sartorius B, Drain PK, Thompson RM. Implementation of Point-of-Care Diagnostics in Rural Primary Healthcare Clinics in South Africa: Perspectives of Key Stakeholders. Diagnostics (Basel). 2017;7(1).

32. Drain PK, Hyle EP, Noubary F, Freedberg KA, Wilson D, Bishai WR, et al. Diagnostic point-of-care tests in resource-limited settings. The Lancet Infectious Diseases. 2014;14(3):239-49.

33. Damschroder LJ, Aron DC, Keith RE, Kirsh SR, Alexander JA, Lowery JC. Fostering implementation of health services research findings into practice: a consolidated framework for advancing implementation science. Implement Sci. 2009;4:50.

For peer review only

For peer review only - http://bmjopen.bmj.com/site/about/guidelines.xhtml

PLUS Healthcare Provider/Administrator Interview Guide

**Administrative Information**
**Participant ID number: PLUS-5 __ __ __**
**Does the interviewee agree to be audio recorded during the interview?** ☐ Yes ☐ No
**Date written informed consent (IC) signed:** |D|D| - |M|M|M| - |Y|Y|Y|Y|
**Consent form signed prior to any study questions?** ☐ Yes ☐ No
**Name of the person who explained the IC:**
**In which language was the IC explained?** ☐ Portuguese ☐ Changana ☐ English
**Date of interview:** |D|D| - |M|M|M| - |Y|Y|Y|Y|
**Location of interview:**
**Was the interview audio-recorded?** ☐ Yes ☐ No
**Start time:** |H|H| : |M|M| **End time:** |H|H| : |M|M|
**Name of interviewer:**

Instruction for research team members

- Use this document as a guide to the interview with a healthcare provider/administrator.
- Conduct the interview in the language with which the interviewee feels most comfortable.
- Prior to beginning the interview, show the healthcare provider/administrator the lung ultrasound probe and tablet. Let the provider/administrator hold and explore the tablet, application and probe.
- Please introduce each question separately. The interview must flow as a conversation. If you notice that the interviewee is hesitant in answering, does not give an in-depth response, or the response is not satisfactory, please probe or ask follow-up questions, but do NOT prompt any specific answer. Several probes are suggested, but you may also ask follow-up questions that are not listed in this guide but are necessary for the complete expression of the interviewee's views.
- Please record the proceedings using the tape recorder/digital recorder (if consent is provided) and state the Participant ID number. Please also record the verbatim responses in appropriate boxes.
- All responses must be treated with confidentiality. Do not discuss or share the responses with anyone outside of the PLUS study team.

A. Demographic information & background

Age:

Gender:

Highest level of education completed:

Years of medical education/training:

Type of medical education/training received (e.g., medical doctor, technician, nurse):

For peer review only - http://bmjopen.bmj.com/site/about/guidelines.xhtml

Duration of employment at [name of facility]:<br><br>Duration of employment in current role:<br><br>Years of medical experience after training completed:
**B.** Role of healthcare provider / administrator
How would you describe your role here at [name of facility]?<br><br>What are your responsibilities?
Did you work in the medical field prior to working here? If yes, please describe.<br>*Probes: length of time worked, responsibilities*
Are you involved in patient care? Please describe your roles and responsibilities.<br><br>Are you involved in clinical research? Please describe your roles and responsibilities.

For peer review only - http://bmjopen.bmj.com/site/about/guidelines.xhtml

For peer review only

Do you ever contribute to the policy development for the care and treatment of children at this facility? Please describe.

C. Environment

What are the most common reasons that caregivers bring their children to [facility name]?

If a caregiver does not bring his/her child here, what are the other options for seeking care?

- Probe for other facilities and other types of healthcare providers such as traditional healers, faith-based healers, drug shops, etc.
- Why might they seek care at another facility or with another type of healthcare provider?

What are the current constraints to providing care to children at [facility name]? Please explain.

- What makes providing care more difficult?
- What makes it easier?

For peer review only

For peer review only - http://bmjopen.bmj.com/site/about/guidelines.xhtml

D. Pneumonia diagnosis

The questions in Part D may not be appropriate for all interviewees. They may be skipped at the discretion of the local principal investigator or designee.

Tell me about the use of the Integrated Management of Childhood Illnesses (IMCI) guidelines at this facility. Do healthcare providers use IMCI? If yes, how do they use them?

If they don't use IMCI, why not? What do they use to guide diagnosis and treatment?

For pneumonia specifically, tell me about the role of IMCI guidelines in diagnosis. If IMCI guidelines are used, please describe. If IMCI guidelines are not used, how is pneumonia diagnosed?

Tell me about the use of chest x-ray at this facility.

Tell me about its role in diagnosing pneumonia.

When are chest x-rays used as a diagnostic tool for children? How is the decision made to order and use a chest x-ray in the care of a child?

For peer review only

For peer review only - http://bmjopen.bmj.com/site/about/guidelines.xhtml

What constraints have providers at [name of facility] faced in using x-ray in caring for children?
**E.** Use of technology
What technologies are used for patient care at [name of facility]? Please describe the technologies and their use.
Are any handheld or portable devices used for patient care at [name of facility]? If so, please describe.<br>• Smartphones?<br>• Tablets?<br>• Other mHealth applications?<br><br>How are these devices stored and maintained?<br>• Are they shared between providers?<br>• How are they kept secure?<br>• How are they cleaned?
Probe on how involved or familiar the respondent is with the PLUS study and its procedures/purpose. Aside from the PLUS study, is ultrasound used in any capacity at [name of facility]?

For peer review only

For peer review only - http://bmjopen.bmj.com/site/about/guidelines.xhtml

IF YES, for what? How frequently?

F. Experience with ultrasound

This section is for clinical care providers only. If the interviewee is not a clinical care provider, please skip to Part G.

Tell me about your experience with ultrasound? Have you performed it? Seen it performed?

If you have performed ultrasound, for what condition/reason? How long have you been performing ultrasound? How frequently do you do ultrasound examinations?

Now, tell me about your experience with **lung** ultrasound. Have you performed it? Seen it performed? Were you familiar with lung ultrasound prior to this study?

If you have performed lung ultrasound, describe your experience doing so.

- How long have you been performing lung ultrasound?
- How frequently do you do lung ultrasound examinations?

For peer review only - http://bmjopen.bmj.com/site/about/guidelines.xhtml

- What has gone well while in performing ultrasound examinations? What has gone poorly? Have any problems occurred during an ultrasound exam?
- What aspects of lung ultrasound are easy to use? What aspects are difficult to use?

If you have performed lung ultrasound, how much time (in minutes) did a typical lung ultrasound examination take? What factors made exams go more quickly? More slowly?

- How much time was spent describing the exam to the caregiver?
- How much time was spent setting up the exam on the tablet?

If you have performed lung ultrasound, did you interpret the lung ultrasound exams? If so, tell me about your experience.

- What was difficult about it?
- What was easy about it? What could have made it easier?

For peer review only - http://bmjopen.bmj.com/site/about/guidelines.xhtml

How much time (in minutes) did it take to interpret a typical lung ultrasound exam?

- What factors made this process go more quickly? More slowly?

If you did not interpret the lung ultrasound exam, why not? Who did the interpretation?

G. Lung ultrasound training

If you have performed lung ultrasound, how much training (hours/days) did you receive? Please describe this training.

- Who provided this training?
- What aspects of lung ultrasound were easy to learn? What aspects were difficult?

If you have not used lung ultrasound previously, do you think that you could learn how to use the device and perform lung ultrasound exams? Please explain.

How much training (hours and/or days) do you think that this would take?

What barriers would there be to learning how to use the ultrasound device?

For peer review only

For peer review only - http://bmjopen.bmj.com/site/about/guidelines.xhtml

**H.** Overall feasibility, usability and acceptability of lung ultrasound
What do you like about the lung ultrasound system as a whole? What do you dislike?<br><br>What do you like about the tablet? What do you dislike?<br>• Screen/display<br><br>What do you like about the application? What do you dislike?<br>• Usability/navigation/interface<br>• Screen/display<br><br>What do you like about the probe? What do you dislike?<br><br>What would you change to improve this ultrasound system?
Do you think that lung ultrasound could be integrated into this facility? What would this look like?<br><br>What would be some of the facilitators of integrating lung ultrasound?

For peer review only

For peer review only - http://bmjopen.bmj.com/site/about/guidelines.xhtml

What would be some of the challenges/barriers?

What needs to happen order to introduce lung ultrasound successfully?

- Probes: Ease of use during a patient visit, integration into the current hospital flow operationally

How do you think that the lung ultrasound would impact your ability to care for children?

- What would be improved by it?
- What would be difficult about it?

How do you think that caregivers would respond to the incorporation of lung ultrasound into care at [name of facility]? Please explain.

For peer review only - http://bmjopen.bmj.com/site/about/guidelines.xhtml

How do you think decision-makers at local, district and national levels would react to a recommendation involving the use of lung ultrasound for pneumonia diagnosis?

Are there scenarios where you think lung ultrasound should not be used? Please explain.

Do you foresee any problems with keeping the ultrasound or tablet in the hospital when not in use?

- Probes: Losing ultrasound probe or tablet, theft

What level of healthcare provider do you think should perform lung ultrasound exams? Please explain.

What level of healthcare provider do you think should interpret lung ultrasound exams? Please explain.

For peer review only

For peer review only - http://bmjopen.bmj.com/site/about/guidelines.xhtml

Do you think that the same person should perform and interpret the lung ultrasound exams? Please explain.
What do you think would be an appropriate price for a lung ultrasound device? Please explain.
**I.** Closing
Do you have any final comments about the ultrasound device that we did not talk about?<br><br>Do you have any comments on the overall PLUS study that we did not talk about?
Do you have any suggestions for other people we should speak to at [name of facility]?
**"Thank you very much for your time and for all of the helpful information you have provided"**

For peer review only

For peer review only - http://bmjopen.bmj.com/site/about/guidelines.xhtml

PLUS Caregiver Qualitative Sub-Study

Administrative information
**Participant ID number: PLUS-1 __ __ __** *(Use child's study ID from the main PLUS study)*
**Date written informed consent (IC) signed:** |D|D| - |M|M|M| - |Y|Y|Y|Y|
**Caregiver consent form signed prior to any study questions?** ☐ Yes ☐ No
**Name of the person who explained the IC:**
**In which language was the IC explained?** ☐ Portuguese ☐ Changana ☐ English
**Relationship to child of the person signing IC:** ☐ Mother ☐ Father ☐ Other, specify:
**Was an impartial witness necessary?** ☐ Yes ☐ No<br>**If YES, did the impartial witness sign the consent form?** ☐ Yes ☐ No
**Does the caregiver agree to be audio recorded** ☐ Yes ☐ No

PLUS Caregiver Direct Observation Guide

**Name of observer:**
**Ultrasound exam start time:** |H|H| : |M|M| **Ultrasound exam end time:** |H|H| : |M|M|
**Instructions for research team members:**<br>• Observe interactions as the study team member explains the ultrasound examination to the caregiver, and during the ultrasound exam itself.<br>• Record observations in the fields below. Any questions and/or comments from the caregiver should be recorded verbatim.<br>• Your role is that of an observer. Avoid interaction with the ultrasound technician or caregiver during the ultrasound exam.<br>• All responses must be treated with confidentiality. Do not discuss or share the responses with anyone outside of the PLUS study team.<br>• Please cross-check the narratives written with the recorded version as a reference, and correct as necessary.
**Approximately how much time (minutes) did research team staff spend explaining the ultrasound exam to the caregiver?**
**Record verbatim any questions/comments by the caregiver during the explanation of the ultrasound exam, as well as the research staff's responses. Provide context as necessary.**

For peer review only - http://bmjopen.bmj.com/site/about/guidelines.xhtml

**How did the child react to the ultrasound exam?** Examples: crying, calm
**Did you observe anything that stood out to you?**

For peer review only

For peer review only - http://bmjopen.bmj.com/site/about/guidelines.xhtml

PLUS Caregiver In-Depth Interview Guide

**Date of Interview:** |D|D| - |M|M|M| - |Y|Y|Y|Y|
**Start time:** |H|H| : |M|M| **End time:** |H|H| : |M|M|
**Name of interviewer:**

Instructions for research team members:

- Use this document as a guide to the interview with the child's caregiver.
- Conduct the interview in the language with which the caregiver feels most comfortable.
- The interview should take place in a quiet place that allows privacy.
- Please introduce each question separately. The interview must flow as a conversation. If you notice that the caregiver is hesitant in answering, does not give an in-depth response, or the response is not satisfactory, please probe or ask follow-up questions, but do NOT prompt any specific answer. Several probes are suggested, but you may also ask follow-up questions that are not listed in this guide but are necessary for the complete expression of the caregiver's views.
- Please record the proceedings using the tape recorder/digital recorder (if caregiver consent is provided) and state the PLUS Participant ID number. Please also record the verbatim responses in the appropriate boxes.
- Please cross-check the narratives written with the recorded version as a reference, and correct as necessary.
- All responses must be treated with confidentiality. Do not discuss or share the responses with anyone outside of the PLUS study team.

A. Background

Caregiver's age:

Caregiver's gender:

Caregiver's highest level of education completed:

Tell me about (name of the index child), who s/he lives with and who is responsible for caring for him/her.

- Household characteristics (physical, family size, main sources of income)
- Parents, siblings characteristics
- Role of respondent in caretaking (if not the father/mother, why)

B. Visit

For peer review only - http://bmjopen.bmj.com/site/about/guidelines.xhtml

Looking at the past 3 months, what was your healthcare use in regard to child health care?

- Probe to help participant estimate his/her use for routine visits, illness episodes, chronic illness follow up, clinical study visits
- Do you consider these (refer to the past three months) as typical for you? (If not, what made this period different from what is usual?)

What makes you decide to bring your child into a health facility?

Do you ever go to another health facility if your child/children get sick? Where is that located?

- Which health facilities did you mostly use?
- Why might you go to another health facility instead of this one?

How long does it take you to get to this health facility? Is there a closer health facility to your home? If so, how long does it take you to get there? Describe the care provided by the facility closest to your home.

C. Acceptability of the tablet

Have you ever seen a healthcare provider use a tablet or smart phone application while caring for your child?

For peer review only

For peer review only - http://bmjopen.bmj.com/site/about/guidelines.xhtml

IF YES: Where? For what condition/reason?
What do you think about the use of tablets as tools to help healthcare providers provide care for children? Please explain.<br><br>Do you think that tablets can be helpful in medical care? Please explain.<br><br>Do you trust tablets as tools to support medical care? Please explain.
**D. Acceptability of the ultrasound device**
Before today, had you ever seen a healthcare provider use an ultrasound device? IF YES, where? For what reason?

For peer review only - http://bmjopen.bmj.com/site/about/guidelines.xhtml

Before today, had a healthcare provider ever used an ultrasound device on your child? IF YES, where? For what reason?
What did you think about the ultrasound device as it was being used on your child?<br>Probes: Ultrasound gel, ultrasound probe, tablet, undressing the child, child cries<br><br>What did you like about it/its use?<br><br>What didn't you like about it/its use?
Did the healthcare provider explain what she or he was doing as s/he was using the ultrasound device? If yes, was this helpful?

For peer review only - http://bmjopen.bmj.com/site/about/guidelines.xhtml

If no, what did you expect the healthcare provider to tell you when s/he was using the ultrasound device?<br><br>What do you think is the purpose of the ultrasound device?<br>• If illnesses are mentioned, probe for the exact name of the illness being targeted?
Did you have any concerns about the ultrasound device being used during your child's visit?
Did you look at the screen while the ultrasound device was being used on your child?<br><br>Why or why not?<br><br>If yes, what did you see? Did you find this helpful?
What did you think about the length of the ultrasound exam?

For peer review only

Do you think that the ultrasound device should be used in this hospital? Please explain.

Do you see any problems with using this ultrasound device on children that come to this hospital? Please explain.

E. Comparison to chest x-ray

Before today's visit, had your child ever had a chest x-ray? *(Research staff to show photo of an x-ray machine)*

What did you think about the use of the ultrasound device compared to the chest x-ray?

What did you like more? Why?

What did you like less? Why?

F. Influence of ultrasound device on care seeking and perceived care

For peer review only - http://bmjopen.bmj.com/site/about/guidelines.xhtml

Do you think that healthcare providers at this hospital should continue to use an ultrasound when evaluating and caring for sick children? Please explain.
If you were choosing between taking your child to a health facility that offers ultrasound and a health facility that does not, would the ultrasound factor into your choice? Please explain.
**G. Closing**
Do you have any final comments about the ultrasound device that we did not talk about?<br><br>Do you have any final comments about the research study that we did not talk about?
**“Thank you very much for your time and for all of the helpful information you have provided”**

For peer review only

For peer review only - http://bmjopen.bmj.com/site/about/guidelines.xhtml

Reporting checklist for qualitative study.

Based on the SRQR guidelines.

Instructions to authors

Complete this checklist by entering the page numbers from your manuscript where readers will find each of the items listed below.

Your article may not currently address all the items on the checklist. Please modify your text to include the missing information. If you are certain that an item does not apply, please write "n/a" and provide a short explanation.

Upload your completed checklist as an extra file when you submit to a journal.

In your methods section, say that you used the SRQRreporting guidelines, and cite them as:

O'Brien BC, Harris IB, Beckman TJ, Reed DA, Cook DA. Standards for reporting qualitative research: a synthesis of recommendations. Acad Med. 2014;89(9):1245-1251.

		Reporting Item	Page Number
**Title**			
	#1	Concise description of the nature and topic of the study identifying the study as qualitative or indicating the approach (e.g. ethnography, grounded theory) or data collection methods (e.g. interview, focus group) is recommended	1
**Abstract**			
	#2	Summary of the key elements of the study using the abstract format of the intended publication; typically includes background, purpose, methods, results and conclusions	2
**Introduction**			
Problem formulation	#3	Description and signifcance of the problem / phenomenon studied: review of relevant theory and empirical work; problem statement	5
Purpose or research question	#4	Purpose of the study and specific objectives or questions	5

For peer review only

Methods

Qualitative approach and research paradigm	#5	Qualitative approach (e.g. ethnography, grounded theory, case study, phenomenolgy, narrative research) and guiding theory if appropriate; identifying the research paradigm (e.g. postpositivist, constructivist / interpretivist) is also recommended; rationale. The rationale should briefly discuss the justification for choosing that theory, approach, method or technique rather than other options available; the assumptions and limitations implicit in those choices and how those choices influence study conclusions and transferability. As appropriate the rationale for several items might be discussed together.	8
Researcher characteristics and reflexivity	#6	Researchers' characteristics that may influence the research, including personal attributes, qualifications / experience, relationship with participants, assumptions and / or presuppositions; potential or actual interaction between researchers' characteristics and the research questions, approach, methods, results and / or transferability	7
Context	#7	Setting / site and salient contextual factors; rationale	6
Sampling strategy	#8	How and why research participants, documents, or events were selected; criteria for deciding when no further sampling was necessary (e.g. sampling saturation); rationale	6-7
Ethical issues pertaining to human subjects	#9	Documentation of approval by an appropriate ethics review board and participant consent, or explanation for lack thereof; other confidentiality and data security issues	8-9
Data collection methods	#10	Types of data collected; details of data collection procedures including (as appropriate) start and stop dates of data collection and analysis, iterative process, triangulation of sources / methods, and modification of procedures in response to evolving study findings; rationale	7
Data collection instruments and technologies	#11	Description of instruments (e.g. interview guides, questionnaires) and devices (e.g. audio recorders) used for data collection; if / how the instruments(s) changed over the course of the study	7

For peer review only

Units of study	#12	Number and relevant characteristics of participants, documents, or events included in the study; level of participation (could be reported in results)	9-10
Data processing	#13	Methods for processing data prior to and during analysis, including transcription, data entry, data management and security, verification of data integrity, data coding, and anonymisation / deidentification of excerpts	7,8
Data analysis	#14	Process by which inferences, themes, etc. were identified and developed, including the researchers involved in data analysis; usually references a specific paradigm or approach; rationale	8
Techniques to enhance trustworthiness	#15	Techniques to enhance trustworthiness and credibility of data analysis (e.g. member checking, audit trail, triangulation); rationale	8
**Results/findings**			
Syntheses and interpretation	#16	Main findings (e.g. interpretations, inferences, and themes); might include development of a theory or model, or integration with prior research or theory	12-20
Links to empirical data	#17	Evidence (e.g. quotes, field notes, text excerpts, photographs) to substantiate analytic findings	12-20
**Discussion**			
Intergration with prior work, implications, transferability and contribution(s) to the field	#18	Short summary of main findings; explanation of how findings and conclusions connect to, support, elaborate on, or challenge conclusions of earlier scholarship; discussion of scope of application / generalizability; identification of unique contributions(s) to scholarship in a discipline or field	20-23
Limitations	#19	Trustworthiness and limitations of findings	23-24
**Other**			
Conflicts of interest	#20	Potential sources of influence of perceived influence on study conduct and conclusions; how these were managed	25
Funding	#21	Sources of funding and other support; role of funders in data collection, interpretation and reporting	25

For peer review only

None The SRQR checklist is distributed with permission of Wolters Kluwer © 2014 by the Association of American Medical Colleges. This checklist can be completed online using https://www.goodreports.org/, a tool made by the EQUATOR Network in collaboration with Penelope.ai

For peer review only
